# Effectiveness of the Ike Dike in mitigating coastal flood risk under multiple climate and sea level rise projections

**DOI:** 10.1111/risa.70060

**Published:** 2025-06-18

**Authors:** Seokmin Son, Chaoran Xu, Meri Davlasheridze, Ashley D. Ross, Jeremy D. Bricker

**Affiliations:** ^1^ Department of Civil and Environmental Engineering University of Michigan Ann Arbor Michigan USA; ^2^ National Engineering Research Center of Port Hydraulic Construction Technology Tianjin Research Institute for Water Transport Engineering, M.O.T. Tianjin China; ^3^ State Key Laboratory of Estuarine and Coastal Research, School of Marine Sciences East China Normal University Shanghai China; ^4^ Department of Marine and Coastal Environmental Science Texas A&M University at Galveston Galveston Texas USA; ^5^ Faculty of Civil Engineering and Geosciences Delft University of Technology Delft The Netherlands

**Keywords:** expected annual damage, flood risk, Ike Dike, probabilistic flood depth, storm surge simulation

## Abstract

In the aftermath of Hurricane Ike in 2008 in the United States, the “Ike Dike” was proposed as a coastal barrier system, featuring floodgates, to protect the Houston‐Galveston area (HGA) from future storm surges. Given its substantial costs, the feasibility and effectiveness of the Ike Dike have been subjects of investigation. In this study, we evaluated these aspects under both present and future climate conditions by simulating storm surges using a set of models. Delft3D Flexible Mesh Suite was utilized to simulate hydrodynamic and wave motions driven by hurricanes, with wind and pressure fields spatialized by the Holland model. The models were validated against data from Hurricane Ike and were used to simulate synthetic hurricane tracks downscaled from several general circulation models and based on different sea level rise projections, both with and without the Ike Dike. Flood maps for each simulation were generated, and probabilistic flood depths for specific annual exceedance probabilities were predicted using annual maxima flood maps. Building damage curves were applied to residential properties in the HGA to calculate flood damage for each exceedance probability, resulting in estimates of expected annual damage as a measure of quantified flood risk. Our findings indicate that the Ike Dike significantly mitigates storm surge risk in the HGA, demonstrating its feasibility and effectiveness. We also found that the flood risk estimates are sensitive to hurricane intensity, the choice of damage curve, and the properties included in the analysis, suggesting that careful consideration is needed in future studies.

## 1 INTRODUCTION

**FIGURE 1:**
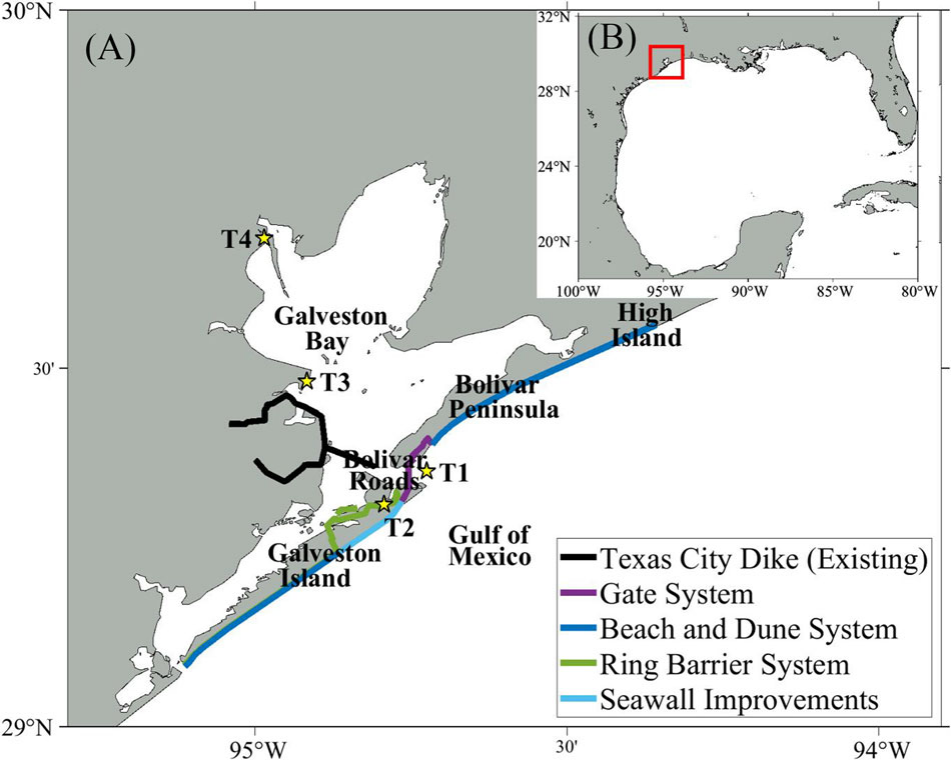
Schematic illustration of the study area: (A) HGA with the recommended plan for the Galveston Bay Storm Surge Barrier System; the yellow stars are NOAA tide stations; (B) Gulf of Mexico; the red box indicates the HGA.

The Houston‐Galveston area (HGA), located in southeast Texas along the upper Gulf Coast (Figure 1), has been severely threatened by storm surges generated by hurricanes originating in the Atlantic Ocean and intensifying as they cross the Gulf of Mexico. One of the most devastating events was in 1900, when a major hurricane struck Galveston Island with a 15 ft storm surge, resulting in approximately 6000 fatalities—the deadliest hurricane in US history (Weems, 1952). This catastrophe led to the construction of the Galveston Seawall, a 17 ft high (relative to mean low water) barrier along the eastern coastline of Galveston Island (USACE, 1981). This seawall significantly reduced hurricane damage on Galveston Island for many years. However, in 2008, Hurricane Ike brought a 10–15 ft (NAVD88) storm surge to Galveston Island and Harris County, Texas, and a 15–20 ft (NAVD88) surge to the Bolivar Peninsula and Chambers County, Texas (Berg, 2009). The resulting damage was extensive, estimated as USD 30 billion (NHC, 2018). Hurricane Ike serves as a warning of the potential for future catastrophic storm surges. Research indicates that global warming is likely to increase hurricane intensities (Emanuel, 2005; Knutson & Tuleya, 2004; Webster et al., 2005) and contribute to sea level rise (SLR), which further raises the vulnerability of coastal regions to storm surge flooding (Frazier et al., 2010; Kleinosky et al., 2007; Tebaldi et al., 2012). Furthermore, future economic growth is expected to amplify hurricane damage costs (Geiger et al., 2016; Mendelsohn et al., 2012), whereas population growth in the HGA will make evacuations increasingly challenging (Merrell et al., 2011).

In response to these threats, there has been a concerted push to implement long‐term safety measures to protect the HGA from future storm surges, particularly those that would repeat the destructive impact of Hurricane Ike. Texas A&M University Galveston proposed the “Ike Dike” concept—a coastal barrier system that would extend the Galveston Seawall to the west of Galveston Island, construct a barrier along the Bolivar Peninsula coastline, and install floodgates at the mouth of Galveston Bay (Merrell et al., 2011). Jonkman et al. (2015) proposed a similar design for a coastal spine system, which includes land barriers and a seawall along the coastlines of Galveston Island and the Bolivar Peninsula, as well as articulated storm surge barriers for navigation and environmental flows between the island and the peninsula. Jackson State University (JSU) investigated different alignments of the Ike Dike by simulating storm surges and found that all alignments significantly reduced surge levels (Ebersole et al., 2018). The US Army Corps of Engineers (USACE) Galveston District proposed comprehensive plans for the protection and restoration of the Texas coast (USACE & GLO, 2021a). These plans include the Galveston Bay Storm Surge Barrier System, which extends from the west of Galveston Island to High Island, consisting of a gate system, beach and dune systems, a ring barrier system, and Galveston Seawall improvements. The gate system is comprised of different types of gates and walls across Bolivar Roads with a crest level of 21.5 ft (NAVD88), and the beach and dune system is designed as a dual dune system with a 14 ft (NAVD88) landward dune and a 12 ft (NAVD88) seaward dune. The ring barrier system encompasses northeast Galveston Island with a crest level of 14 ft (NAVD88), and improvements of the existing seawall along the Galveston coastline raise its crest level to 21 ft (NAVD88). For these USACE's plans, a USD 31 billion bill was authorized by the US Congress, marking the largest civil undertaking by the USACE (Douglas, 2022). The schematic design of the Ike Dike is illustrated in Figure 1.

The Ike Dike project has faced scrutiny regarding its feasibility. Economic assessments indicate that the benefits of the coastal spine outweigh its costs, supporting the practicability of the barrier system for mitigating storm surge risks (Davlasheridze et al., 2019; Davlasheridze et al., 2021). Additionally, policies advocating for the construction of seawalls and levees or the rehabilitation of dunes have garnered strong support from residents of the HGA, particularly those who have experienced coastal flood damages or perceive higher flood risks (Ross & Atoba, 2022). However, these studies assumed current climate conditions and sea level, so further research is necessary to determine whether the Ike Dike would remain effective under future climate and sea level scenarios. Concerns have also been raised about the effectiveness of the Ike Dike in protecting the region against major hurricanes (Bittle, 2023; Keller, 2024; Peters, 2024). Critics argue that the coastal spine may not effectively withstand Category 4 or 5 hurricanes (Simpson, 1974), which can produce storm surges of 25 ft (NAVD88) or higher (Blackburn, 2019). Moreover, the parallel dune system with heights of 14 and 12 ft (NAVD88) may be inadequate to protect Galveston Island and the Bolivar Peninsula from severe hurricanes (Merrell, 2021). The USACE's design includes a shorter western barrier of within the dune system, compared to the earlier barrier designs proposed by Jonkman et al. (2015) and JSU, to allow water flow into the bay. This modification, however, could allow fore‐runner surge in Galveston Bay and prevent the bay from being sealed at low tide (Merrell, 2021).

**FIGURE 2:**
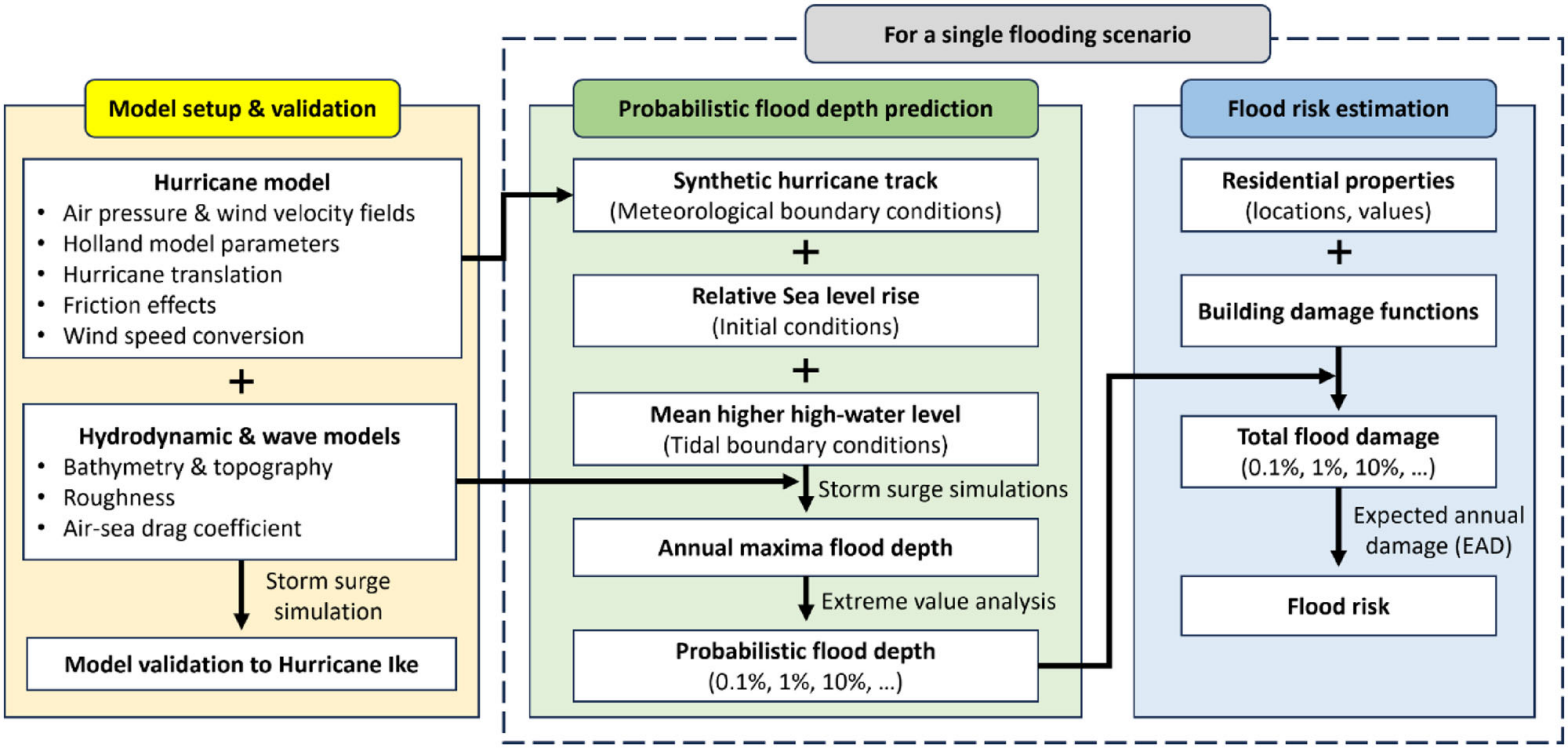
Flowchart of the study.

In this study, we evaluate the long‐term feasibility and effectiveness of the proposed Ike Dike under present and end‐of‐century climate scenarios by quantifying coastal flood risk in the HGA. The coastal flood risk associated with storm surges strongly depends on storm intensity and SLR (Woodruff et al., 2013). To account for different scenarios, we conduct numerical simulations under several sets of synthetic hurricane tracks, both without and with the Ike Dike, for present and future climate scenarios and different SLR projections in storm surge models. Prior to the simulations, a hydrodynamic model, a wave model, and a hurricane wind and pressure model are validated using data from Hurricane Ike. The simulated flood depths are used to predict probabilistic flood depths for specific annual exceedance probabilities. A common method to relate flood damage to residential buildings to flood depth is through a damage function (Suppasri et al., 2013). By applying several damage functions to residential properties in the HGA, we estimate flood risk under different flooding scenarios to evaluate the effectiveness of the Ike Dike. Furthermore, as our study takes into account several input parameters, including climate models, SLR, and damage functions, we evaluate the robustness of storm surge risk and the performance of the Ike Dike with respect to these parameters. This approach aims to identify the significant factors to be considered in future coastal flood risk studies. Figure 2 illustrates the flowchart of the study that highlights the specific objectives mentioned above.

The remainder of the article is structured as follows. Section 2 details the materials and methods used in this study, and Section 3 describes the results of the probabilistic flood depth predictions and flood risk estimations. These findings are discussed in Section 4, and the overall study is summarized and concluded in Section 5.

## 2 MATERIALS AND METHODS

### 2.1 Model setup and validation

#### 2.1.1 Hydrodynamic and wave model domains and setup

For conducting our storm surge simulations, the Delft3D Flexible Mesh (D‐Flow FM) suite was selected (Deltares, 2024a). D‐Flow FM solves the unsteady shallow water equations for an incompressible fluid over an unstructured grid, which allows for flexible and detailed resolution of complex geometries. To capture the interactions between wave dynamics and current flows, D‐Flow FM is integrated with the third‐generation spectral wave model Simulating Waves Nearshore (SWAN) (Deltares, 2024b). SWAN works on a structured grid and solves the spectral action balance equation responsible for the generation, propagation, and dissipation of waves.

**FIGURE 3:**
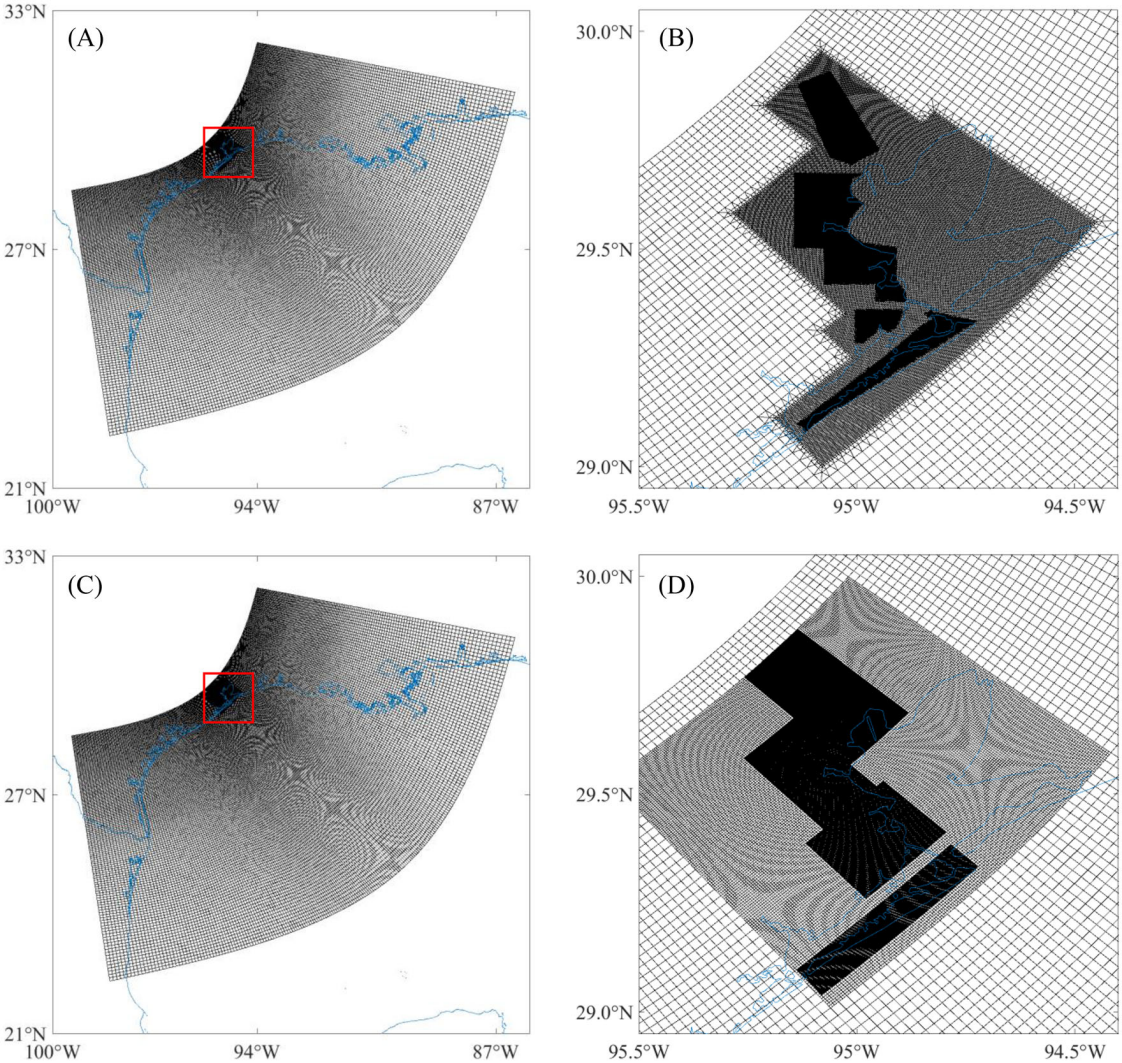
Domain and grid structures of the models, where blue indicates the coastline: (A) full extent of the domain and grid of the flow model, the red box indicates the refined grid; (B) the refined grid of the flow model in the HGA; (C) full extent of the domain and grid of the wave model, the red box indicates the nested grids; (D) the nested grids of the wave model in the HGA.

The domain of the model encompasses the northwest region of the Gulf of Mexico, geographically ranging from 22 to 32°N and from 100 to 86°W. Within the flow model, the grid consists of approximately 280,000 cells of varying resolutions to accurately capture different scale features. The grid resolution is finer in the HGA, with cells sized 60 m × 60 m, whereas further offshore, the resolution is coarser, with cell sizes of 5 km × 10 km. The grid of the wave model is distinct from that of the flow model and consists of multiple nested grids. The outermost grid covers the full extent of the modeling domain, matching the flow model's spatial reach. Nested within this, the intermediate nested grid covers the HGA with a resolution of 500 m × 500 m, and the smallest grid focuses on the coastline and Galveston Island with a finer resolution of 150 m × 150 m (Xu et al., 2023). Figure 3 illustrates the domain and grid structures of the flow and wave models.

Model stability is a critical concern, and setting a proper simulation time step is essential for maintaining it. The flow model has a time step automatically adjusted to keep the maximum Courant number less than 0.5, whereas the wave model is run in its stationary mode and updates hourly. The coupling of the flow and wave models takes place at intervals of 1 h, allowing for interaction between models without compromising the stability or accuracy of the simulations.

We sourced global bathymetry from the General Bathymetric Chart of the Oceans (GEBCO) (GEBCO Compilation Group 2023), providing a 15‐arcsec spatial grid (GEBCO, 2023). For high‐resolution topography data in the HGA, we used the National Centers for Environmental Information (NCEI) Coastal Relief Model with a 1‐arcsec grid (NOAA, 2023). For depicting variations in land and ocean roughness in the flow model, Manning's *n* coefficients are derived according to land cover classifications from the National Land Cover Database (NLCD) (Bunya et al., 2010; Dewitz, 2023). In addition, the air–sea drag coefficient is crucial for both storm surge and wave prediction (Charnock, 1955). In the study, we compared the air–sea drag coefficients of Makin (2005) and Zweers et al. (2010), then adopted Zweers et al. (2010)’s one. For scenarios that include the Ike Dike barriers, these structures are implemented as fixed weirs in D‐Flow FM; these prohibit flow exchange between the two adjacent cells up to an assigned crest level.

#### 2.1.2 Hurricane model

To simulate the hurricanes in the hydrodynamic model, hurricane track data are spatialized into spiderweb pressure and wind velocity fields to serve as input data to the storm surge and wave models. Using Holland's model (Holland, 1980), the air pressure and tangential wind velocity fields are computed as follows:

(1) $$P\;\left(r\right)=P_{c}+\left(P_{n}-P_{c}\right)\exp\left(-\frac{A}{r^{B}}\right),$$

(2) $$V_{g}\;\left(r\right)=\sqrt{\frac{AB\left(P_{n}-P_{c}\right)\exp\left(-\frac{A}{r^{B}}\right)}{\rho r^{B}}+\frac{f^{2}r^{2}}{4}}\;-\frac{fr}{2},$$

where P(r) is surface pressure at a distance r from the center of hurricane, $P_{c}$ is central surface pressure, $P_{n}$ is ambient surface pressure (= 1015 hPa), $V_{g}\left(r\right)$ is tangential wind velocity at a distance r from the center of the hurricane, ρ is density of air (= 1.2 kg/m^3^), f is the Coriolis parameter, and A and B are Holland model parameters. By comparing the accuracy of the hurricane models using different methods of estimating model parameters (Vickery & Wadhera, 2008; Willoughby & Rahn, 2004), the parameters are estimated by Holland et al. (2010) as follows:

(3) $$A={\left(R_{\max}\right)}^{B}\;,$$

(4) $$B=\frac{\rho e{\left(V_{\max}\right)}^{2}}{P_{n}-P_{c}}\;,$$

where $R_{\max}$ is the radius of maximum sustatined wind, e is the Euler number, and $V_{\max}$ is maximum 10‐m ground relative wind speed. As $V_{g}\left(r\right)$ cannot represent the hurricane's asymmetric structure due to the interaction with steering flow caused by hurricane translation, the original Holland model was improved to represent the translation. Xie et al. (2006) is used to implement new wind vector, which achieves better accuracy than rescaling $V_{g}\left(r\right)$ with any additional coefficient (Kalourazi et al., 2020):

(5) $$v\left(r,\theta \right)=V_{g}\;\left(r,\theta \right)+0.5V_{t},$$

(6) $$V_{g}\;\left(r,\theta \right)=\left(\begin{array}{c} V_{g}\left(r\right)\cos\left(\theta +\beta \right)\\ V_{g}\left(r\right)\sin\left(\theta +\beta \right) \end{array}\right)\;,\;V_{t}=\left(\begin{array}{c} V_{t}\cos\alpha \\ V_{t}\sin\alpha \end{array}\right),$$

where v is the wind velocity vector at a distance r from the center of the hurricane and at an azimuth of θ, $V_{t}$ is the hurricane translation speed, β is the angle of inflow, and α is the angle from the direction of the hurricane translation. β represents the friction effects caused by hurricane translation obtained as follows (Graham & Nunn, 1959):

(7) $$\beta \left(r\right)=\left\{\begin{array}{cc} \frac{10r}{R_{\max}}, & r<R_{\max}\\ 10+75\left(\frac{r}{R_{\max}}-1\right), & R_{\max}\leq r<1.2R_{\max}\\ 25, & r\geq 1.2R_{\max} \end{array}\right.$$

For storm surge simulations, it is necessary to use at least 10‐min average wind speeds, as these provide a more stable estimate of the wind's force overtime compared to shorter averaging periods (Deltares, 2024c). The 1‐min average wind speeds from our track data were converted to 10‐min average wind speeds using a gust factor, which is a numerical value that represents the ratio between the peak wind gust over a specific duration and the average wind speed for a period of time (Krayer & Marshall, 1992). The conversion factor from 1 to 10‐min average wind speeds is determined by dividing the 10‐min gust factor (1.08) by the 1‐min gust factor (1.32). As a result, we get a conversion factor of 0.818, which must be applied to $V_{\max}$ for each hurricane so that the equivalent 10‐min average wind speed can be estimated.

#### 2.1.3 Model validation to Hurricane Ike

**FIGURE 4:**
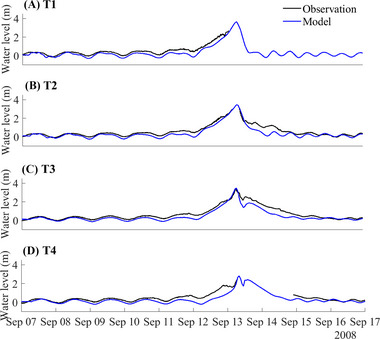
Comparison of the modeled water level to the observed water level during Hurricane Ike at different tide stations (relative to MSL): (A) T1 (8771341 Galveston Bay Entrance); (B) T2 (8771450 Galveston Pier 21); (C) T3 (8771013 Eagle Point); (D) T4 (8770613 Morgans Point).

**FIGURE 5:**
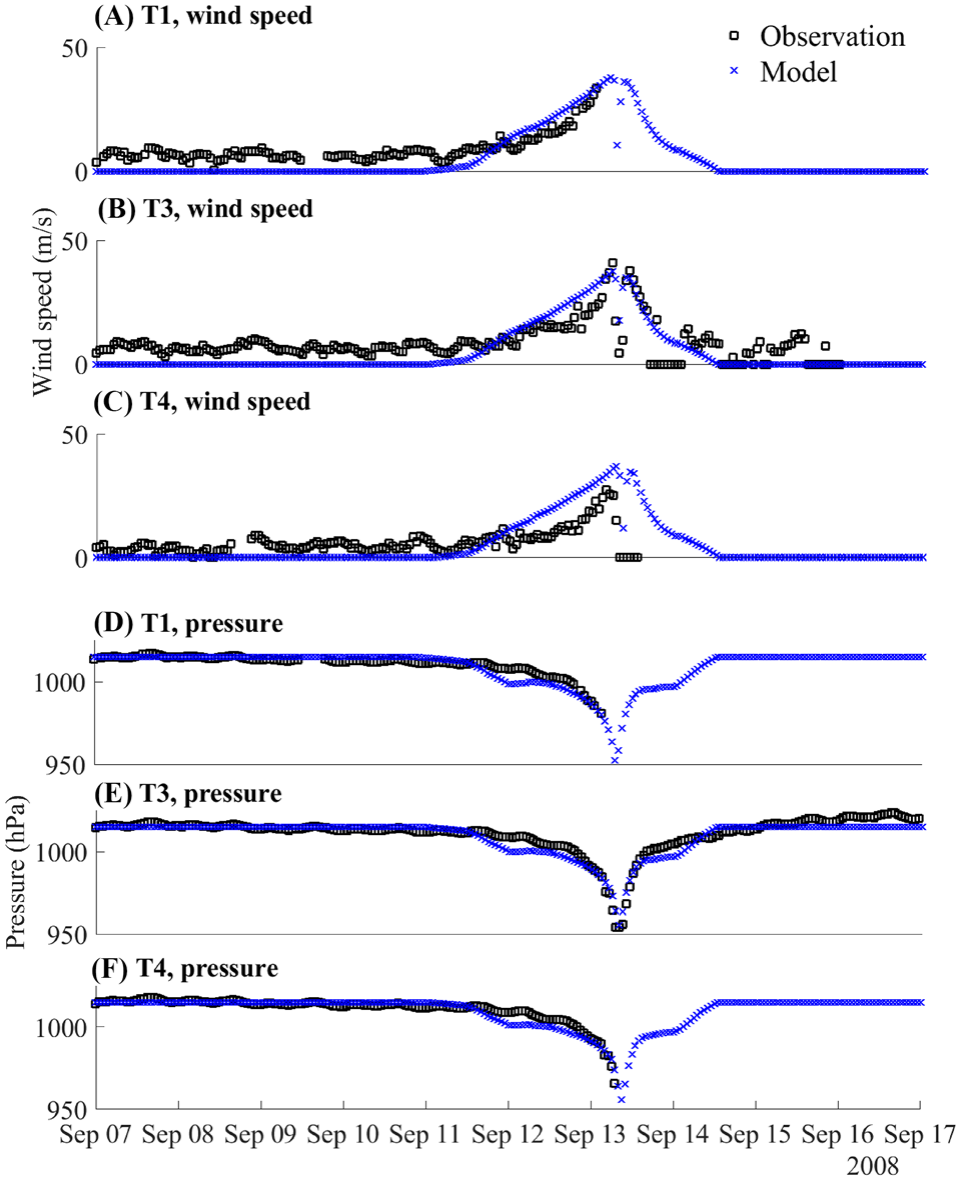
Comparison of the modeled wind speed or pressure to the observed wind speed (converted to 10‐min average 10‐m ground wind speed) and pressure during Hurricane Ike at different tide stations: (A) wind speed at T1 (8771341 Galveston Bay Entrance); (B) wind speed at T3 (8771013 Eagle Point); (C) wind speed at T4 (8770613 Morgans Point); (D) pressure at T1; (E) pressure at T3; (F) pressure at T4.

**FIGURE 6:**
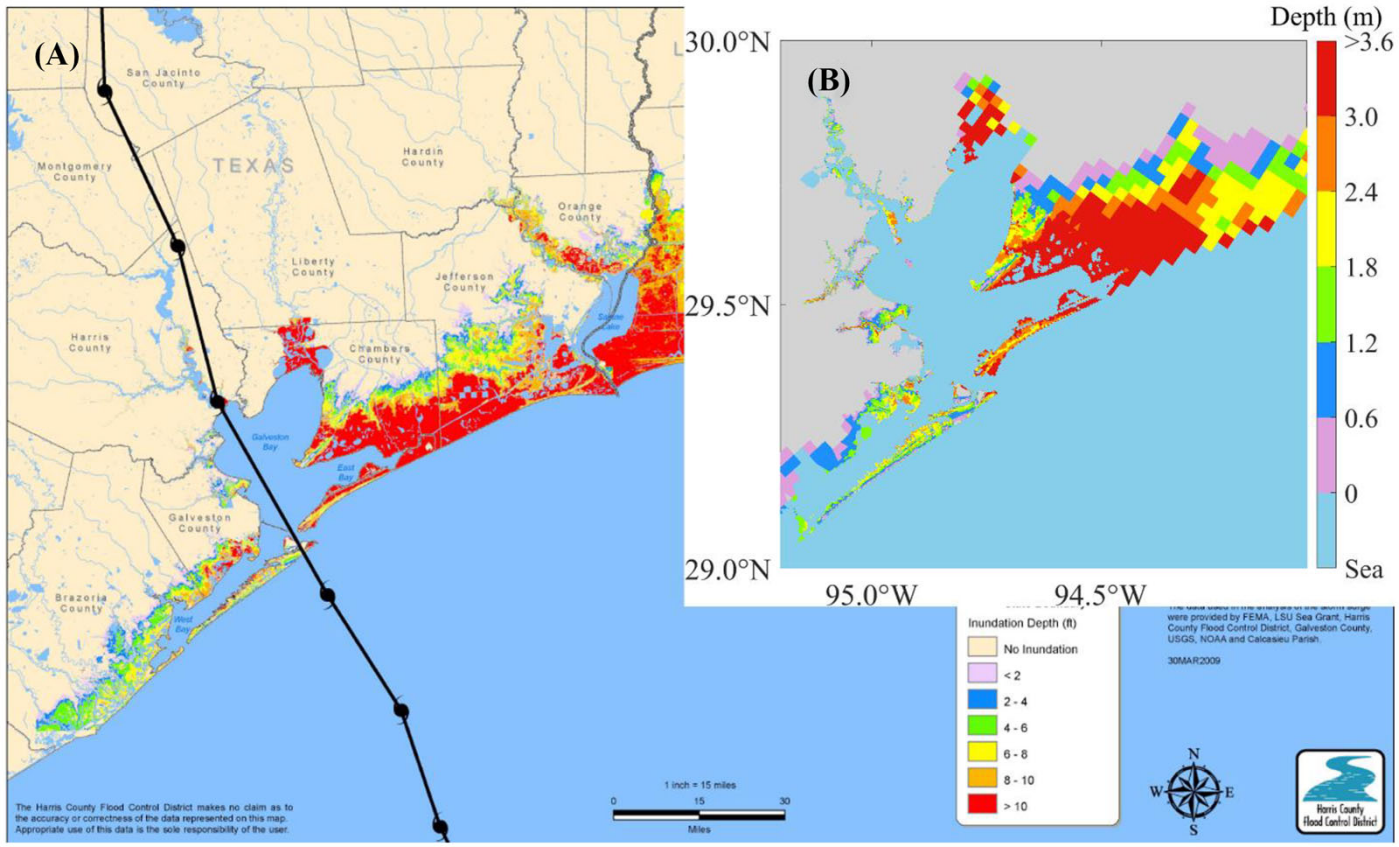
Comparison of the maximum modeled inundation depth to the observed inundation depth map during Hurricane Ike: (A) observed flood depths (HCFCD, 2009) (B) simulated flood depths.

To ensure the reliability and accuracy of our models, we validated the model by comparing the model outputs against water level (Figure 4), wind speed, and pressure (Figure 5) observed during Hurricane Ike, as well as overland inundation extent and depth (Figure 6). The tropical cyclone extended best track dataset (EBTRK) from Regional and Mesoscale Meteorology Branch (RAMMB) was used for the meteorological boundary condition input (Demuth et al., 2006). We validated model results against observed data from NOAA tide stations around Galveston Bay: 8771341 Galveston Bay Entrance (T1), 8771450 Galveston Pier 21 (T2), 8771013 Eagle Point (T3), and 8770613 Morgans Point (T4), which are illustrated in Figure 1. Wind speed and pressure were also compared against observations from T1, T3, and T4. The observed wind data recorded were at 1.5 m above the ground and provided as 5‐min averages, needing a conversion to make it compatible with the model outputs. Using Krayer and Marshall (1992) and Allen et al. (1998), the data was converted to 10‐min average wind speed at 10 m above the ground. Simulated inundation depth was compared to the Hurricane Ike inundation depth map created by the Harris County Flood Control District (HCFCD) (HCFCD, 2009). When simulating storm tide conditions for Hurricane Ike within the model, we applied astronomical tide data on the open boundary derived from regional and local models provided by OSU Tidal Inversion Software (OTIS) for the Gulf of Mexico with a 1/45° resolution (Egbert & Erofeeva, 2002). The performance of the model was evaluated using statistical measured of relative root‐mean‐square error (RRMSE) and *R*‐squared values, described in Table 1. RRMSE is calculated as:

(8) $$\mathrm{RRMSE}=\sqrt{\frac{1}{N}\frac{\sum_{i=1}^{N}{\left(x_{i}-{\hat{x}}_{i}\right)}^{2}}{\sum_{i=1}^{N}{\left({\hat{x}}_{i}\right)}^{2}},}$$

where N is the length of data, $x_{i}$ is i‐th modeled data, and ${\hat{x}}_{i}$ is i‐th observed data. These demonstrate strong agreement between modeled and observed data, affirming the reliability of the simulations conducted within our study.

**TABLE 1:** Goodness of fit for modeled water level, wind speed, and pressure compared to the observed data at different tide stations during Hurricane Ike.

Parameter	Station	RRMSE	*R* ^2^
Water level	T1	0.0321	0.9343
	T2	0.0244	0.8707
	T3	0.0200	0.9194
	T4	0.0387	0.7373
Wind speed	T1	0.0509	0.8365
	T3	0.0412	0.7613
	T4	0.0637	0.7651
Pressure	T1	0.0029	0.8463
	T3	0.0025	0.9141
	T4	0.0026	0.8740

Abbreviations: RRMSE, relative root‐mean‐square error.

### 2.2 Probabilistic flood depth predictions

#### 2.2.1 Climate scenarios and synthetic hurricane tracks

For this study, we utilized sets of synthetic hurricane tracks statistically downscaled from three different CMIP6 general circulation models (GCMs) under present and projected future climates by WindRiskTech (Emanuel et al., 2008). The GCMs used include the Canadian Earth System Model (CanESM), the Geophysical Fluid Dynamics Laboratory (GFDL) model, and the Hadley Centre Global Environmental Model (HadGEM). From each GCM, the synthetic dataset comprised 4500 hurricane tracks spanning a period of 30 years. The present dataset covers the years 1981–2010 based on the simulations of 20th century climate, whereas the future dataset is for 2071–2100 under the Intergovernmental Panel on Climate Change's (IPCC's) very high greenhouse gas (GHG) emission scenario SSP5‐8.5 (Shared Socio‐economic Pathway) (IPCC, 2023). Each track in the dataset contains data at 2‐h intervals, including information on the cyclone's geographical position (latitude and longitude), central surface pressure, maximum 10‐m ground relative wind speed, and the radius of maximum winds.

As limitations of our available computing facilities made it impractical to simulate all 4500 hurricane tracks, we narrowed down our focus to those cyclones that could have a major impact on the HGA. To select the relevant cyclone tracks for simulation, two main filtering criteria were considered: landfall location and storm intensity. First, only the tracks are included where the hurricanes made landfall to the west of the longitudinal line 93.78°W, which is approximately 50 km east of Galveston Bay. This ensured that the simulated storms were ones that could affect the HGA. Then, the Saffir–Simpson Hurricane Wind Scale was used to categorize the storms based on their maximum wind speeds (Simpson, 1974). We focused on major hurricanes, which are defined as Category 3 (with wind speeds of 111–129 mph), Category 4 (130–156 mph), and Category 5 (157 mph or higher). Only hurricanes that reached these thresholds when positioned north of 27.5°N, in proximity to Galveston Bay, were evaluated with hydrodynamic simulations. There are also other important metrics to be considered in selecting cyclones, such as angle of approach, radius of maximum winds, and hurricane translation speed (Chouinard et al., 1997; FEMA, 2008). The synthetic hurricane tracks applied here account for a large range of variation in each of these metrics. However, the research team lacked sufficient computational power to simulate storm surge for all synthetic tracks in the database, so a choice we made to only consider major hurricanes (Category 3, 4, or 5) that make landfall with 50 km of HGA.

**FIGURE 7:**
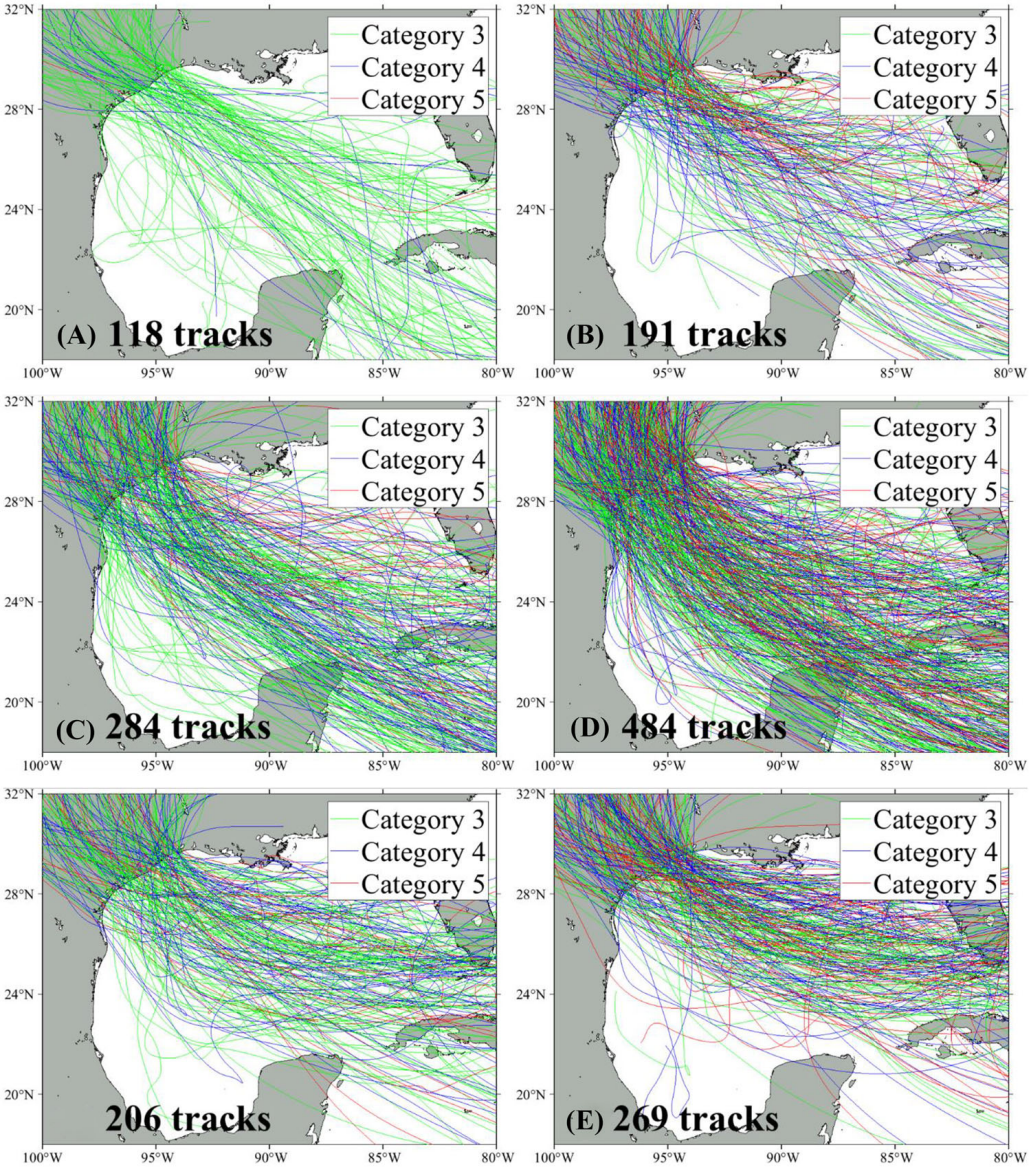
Illustrations of the synthetic tracks selected for simulations with the number of tracks under different climate scenarios: (A) CanESM for the present climate; (B) CanESM for the future climate; (C) GFDL‐6.0 for the present climate; (D) GFDL‐6.0 for the future climate; (E) HadGEM‐6.0 for the present climate; (F) HadGEM‐6.0 for the future climate.

As a result, 118 synthetic tracks were selected for the CanESM under the present climate scenario and 191 tracks under the future climate scenario. For the GFDL model, 284 and 484 tracks were selected for the present and future climate, respectively. For the HadGEM, 206 tracks were used for the present climate and 269 tracks for the future climate. Figure 7 illustrates the synthetic tracks and the number of tracks for each climate scenario. In order to consider the potential impacts of back surge, all the hurricanes were simulated until they dissipated over land, which occurred after they passed over Galveston Bay.

#### 2.2.2 Relative sea level rise

In future climate scenarios, SLR is considered one of the most significant factors affecting coastal areas, making them increasingly vulnerable to the impacts of storm surges (Yang et al., 2014). There are two components to SLR: global SLR and regional SLR. The cumulative effect, which is known as relative SLR, is determined by summing the global and regional SLRs.

Global SLR is predominantly driven by global warming, which contributes to rising sea levels through mechanisms such as the melting of ice sheets and glaciers, as well as the thermal expansion of seawater (Sweet et al., 2022). The extent of future global SLR strongly depends on GHG emission scenarios. To align with the scenarios conceived in our GCMs, we assume a very high GHG emission scenario. According to IPCC (2023), under this scenario, the global SLR relative to the baseline period of 1995–2017 is projected to be 0.20–0.29 m by 2050, escalating to 0.63–1.01 m by 2100. For the purposes of extreme value analysis and flood mapping across specific return periods, a constant SLR must be presumed for the future storm events in 2071–2100. Therefore, a steady global SLR is assumed corresponding to the estimate for the year 2085, which is the midpoint of the 30‐year period to be used in the extreme value analysis as a consistent SLR value, projected to be 0.48–0.75 m. Three global SLR scenarios are utilized for the research: 0.48 m (Scenario 1), 0.57 m (Scenario 2), and 0.75 m (Scenario 3).

On the other hand, regional SLR is influenced by changes in the ocean's circulation patterns and density, alterations in Earth's gravity and rotation, and vertical land movements, including both subsidence and uplift (Sweet et al., 2022). Therefore, it has to be evaluated separately from global SLR. According to (Sweet et al., 2022), regional SLR in the HGA relative to 2000 is projected to reach 0.6 m by 2100 and 0.9 m by 2150. By interpolation, we estimate that the regional SLR by 2085 would be approximately 0.51 m. Considering both global and regional SLR projections, three relative SLR scenarios are constructed for our research: 0.99 m (Scenario 1), 1.08 m (Scenario 2), and 1.26 m (Scenario 3), which are the projected relative SLR by 2085. These scenarios are instrumental in evaluating the potential risks and impacts of storm surge on coastal regions in the face of climate change.

Each flooding scenario is constructed by combining an SLR projection, a GCM, and either the presence or absence of the Ike Dike barriers (Table 2).

**TABLE 2:** Flooding scenario numbers under different general circulation models (GCMs), sea level rise (SLR) projections, and the presence or absence of the Ike Dike barriers, where X means “without the Ike Dike” and O means “with the Ike Dike.”

Present/Future	SLR scenario	GCM	Ike Dike	Flooding scenario #
Present	–	CanESM	X	1
			O	2
		GFDL‐6.0	X	3
			O	4
		HadGEM‐6.0	X	5
			O	6
Future	SLR Scenario 1 (0.99 m)	CanESM	X	7
			O	8
		GFDL‐6.0	X	9
			O	10
		HadGEM‐6.0	X	11
			O	12
	SLR Scenario 2 (1.08 m)	CanESM	X	13
			O	14
		GFDL‐6.0	X	15
			O	16
		HadGEM‐6.0	X	17
			O	18
	SLR Scenario 3 (1.26 m)	CanESM	X	19
			O	20
		GFDL‐6.0	X	21
			O	22
		HadGEM‐6.0	X	23
			O	24

Abbreviations: CanESM, Canadian Earth System Model; GFDL, Geophysical Fluid Dynamics Laboratory; HadGEM, Hadley Centre Global Environmental Model.

#### 2.2.3 Tidal boundary conditions

This study assumes a constant tide level representing the mean higher high water (MHHW) condition (Ke et al., 2021), which is the average of the highest tidal levels recorded daily over a 19‐year period, as observed at tide stations (NOAA, 2000). This assumption implies that the MHHW occurs simultaneously at all locations across our model, simplifying the baseline water level against which storm surges are evaluated. To determine the MHHW level specific to the HGA, the observed MHHW from nearby tide stations was arithmetically averaged, resulting in a tidal level of 0.18 m relative to mean sea level (MSL). This MHHW level is then consistently added to the initial water level in our model, which operates at a datum of MSL. For the present climate scenarios, the initial water level is set as 0.18 m, reflecting the MHHW without additional SLR. Considering relative SLR projections and MHHW, the initial water level for simulations is adjusted to 1.17, 1.26, and 1.44 m for SLR Scenarios 1, 2, and 3, respectively. As in the setup for validation, to mitigate long wave reflections at the model boundaries, we employ a weakly reflective Riemann boundary condition (Deltares, 2024a).

#### 2.2.4 Extreme value analysis

By simulating storm surges for each scenario using the hydrodynamic model, we produce 30‐year annual flood maps that present the maximum flood depths experienced at every location within a given year. From the annual maximum flood depths, we then generate maps representing specific annual exceedance probability floods. To achieve this, extreme value analysis is conducted using probability distribution functions, allowing us to estimate the flood depths associated with specific return periods at each computational cell of the storm surge model. The process is summarized as follows (Bedient et al., 2019):

1. Rank the annual flood depths recorded over 30 years for each model grid cell in descending order. If there are $n_{0}$ zeros out of 30 (=n) data, calculate the discrete probability of zero depth occurrence ($P_{0}$) as $P_{0}=n_{0}/n$.
2. Fit the non‐zero flood depth data to an appropriate probability distribution. The maximum likelihood estimation (MLE) method is employed to derive the parameters of the distribution. Denote the cumulative distribution function (CDF) of this distribution function of non‐zero data as $F_{X,0}$.
3. Adjust the total probability within the distribution to reflect the probability of non‐zero events, scaling the overall probability mass to ($1-P_{0}$) instead of 1. The base value of the CDF is set to $P_{0}$ to account for the occurrence of zero‐depth events. The CDF for the full dataset ($F_{X}$) is geven by:
(9) $$F_{X}\;\left(x\right)=\left\{\begin{array}{cc} P_{0}, & x=0\\ P_{0}+\left(1-P_{0}\right)F_{X,0}\left(x\right), & x>0 \end{array}\right.$$
4. Match $F_{X}$ to the theoretical plotting position ($F_{m}$) which calculates the expected CDF value for each rank m given by Gringorten (1963):
(10) $$F_{m}=1-\frac{m-0.4}{n+1-0.8}.$$

The goodness of fit of the probability distribution is evaluated using root‐mean‐square error (RMSE) and the *R*‐square values. These metrics assess the accuracy and the strength of the fit.

1. Compute the flood depth for a given return period.

The generalized extreme value (GEV) distribution probability distribution is adopted in this study. The GEV distribution is particularly suitable for our purpose because of its flexibility and its widespread adoption in extreme value analysis (Ke et al., 2018; Loaiza et al., 2022; van den Brink et al., 2003), and it has been widely used in significant projects, such as Federal Emergency Management Agency's (FEMA's) flood map project (FEMA, 2023). The CDF of the GEV distribution is expressed as follows:

(11) $$F_{X,0}\left(x\right)=\left\{\begin{array}{cc} \exp\left[-{\left\{1+\xi \left(\frac{x-\mu }{\sigma }\right)\right\}}^{-\frac{1}{\xi }}\right], & \xi \neq 0,1+\xi \left(\frac{x-\mu }{\sigma }\right)>0\\ \exp\left\{-\exp\left(-\frac{x-\mu }{\sigma }\right)\right\}, & \xi =0 \end{array}\right.,$$

where ξ is a shape parameter, μ is a location parameter, and σ is a scale parameter. The value of ξ determines the form of the distribution: Frechet distribution for ξ>0, Gumbel distribution for ξ=0, and inverse Weibull distribution for ξ<0. In our extreme value analysis, there was an issue of excessively high flood depth estimates for the 100 and 500‐year return periods while using the Frechet distribution, corresponding to a positive value of ξ. To achieve a more realistic estimate of flood depths, our approach is to constrain the value of ξ to 0 if it is positive. These forces provide a more practical fit for the flood depth data and preventing overestimation of the flood risk for extreme events. The use of this distribution is justified by comparing its goodness of fit to that of the Gumbel and Weibull distributions, both of which are also recommended for flood map projects by FEMA (FEMA, 2023). Comparison of the results is shown in Table 3.

**TABLE 3:** Comparison of goodness of fit for cumulative distribution functions (CDFs) of the flood depth compared to the theoretical plotting position using the mean of root‐mean‐square errors (RMSEs) and *R* ^2^s at all computational cells.

Flooding scenario #	Gumbel		Weibull		GEV	
	RMSE (m)	*R* ^2^	RMSE (m)	*R* ^2^	RMSE (m)	*R* ^2^
1	0.0575	0.9179	0.0462	0.9419	0.0350	0.9627
2	0.0432	0.9348	0.0356	0.9476	0.0305	0.9596
3	0.0365	0.9479	0.0356	0.9511	0.0314	0.9569
4	0.0362	0.9522	0.0385	0.9521	0.0305	0.9637
5	0.0324	0.9495	0.0328	0.9497	0.0297	0.9526
6	0.0351	0.9431	0.0351	0.9456	0.0303	0.9581
7	0.0528	0.9469	0.0422	0.9635	0.0362	0.9724
8	0.0428	0.9322	0.0410	0.9361	0.0363	0.9437
9	0.0458	0.9587	0.0375	0.9692	0.0351	0.9745
10	0.0435	0.9381	0.0394	0.9430	0.0380	0.9446
11	0.0473	0.9464	0.0402	0.9589	0.0402	0.9591
12	0.0339	0.9333	0.0373	0.9314	0.0324	0.9451
13	0.0535	0.9461	0.0427	0.9632	0.0362	0.9727
14	0.0435	0.9358	0.0418	0.9358	0.0366	0.9449
15	0.0463	0.9594	0.0380	0.9696	0.0355	0.9746
16	0.0440	0.9394	0.0394	0.9452	0.0386	0.9457
17	0.0426	0.9556	0.0423	0.9582	0.0378	0.9634
18	0.0340	0.9306	0.0375	0.9285	0.0327	0.9446
19	0.0552	0.9442	0.0440	0.9622	0.0364	0.9733
20	0.0443	0.9378	0.0423	0.9403	0.0363	0.9521
21	0.0472	0.9596	0.0389	0.9698	0.0360	0.9749
22	0.0440	0.9423	0.0388	0.9486	0.0388	0.9485
23	0.0427	0.9586	0.0433	0.9595	0.0379	0.9657
24	0.0352	0.9339	0.0385	0.9327	0.0338	0.9472

Abbreviation: GEV, generalized extreme value.

### 2.3 Flood risk estimations

#### 2.3.1 Property damages and damage curves

**FIGURE 8:**
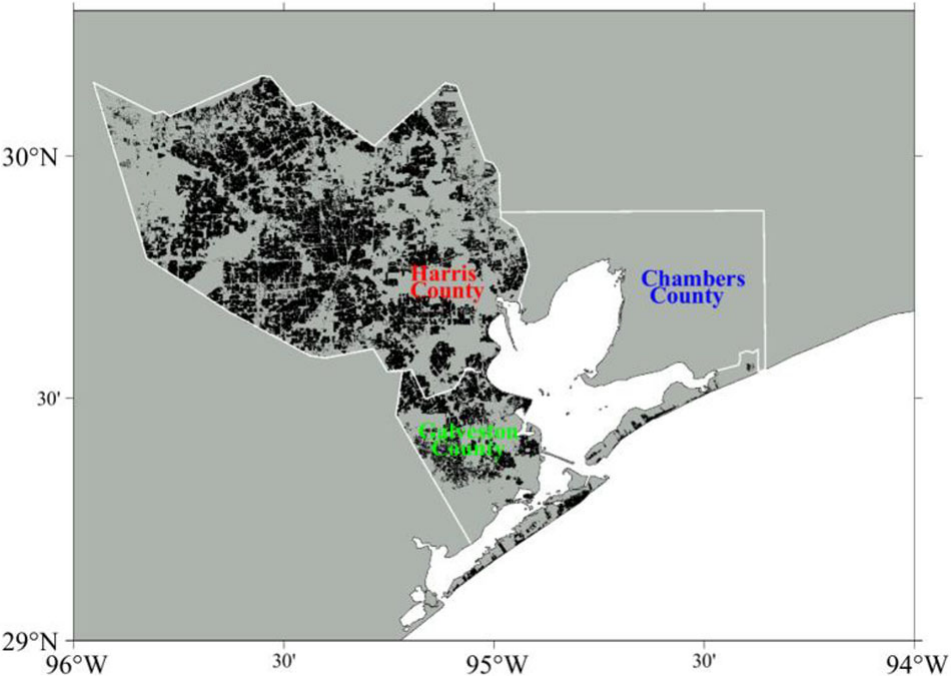
Illustration of the locations of residential properties (black dots) included in the damage analysis.

In advance of estimating the flood risk for each flooding scenario, we compute the flood damages for different annual exceedance probability floods. This study focuses on the total flood damage, which refers to the cumulative damage to residential properties in the HGA, specifically in Harris and Galveston Counties, TX. For this analysis, we utilize the database of CoreLogic, Inc., which includes the locations (latitude and longitude at the parcel level) and assessed values of residential properties as of 2021. The dataset used in the study comprises 1,243,195 residential properties in Harris County and 132,029 in Galveston County, amounting to 1,375,224 properties considered for flood damage assessment, regardless of the property's elevation or material. Among these properties, 1,058,642 properties are owner‐occupied, 281,674 are owner‐absent, and remaining properties are unidentified. Figure 8 illustrates these properties on a gridded map of the HGA.

**FIGURE 9:**
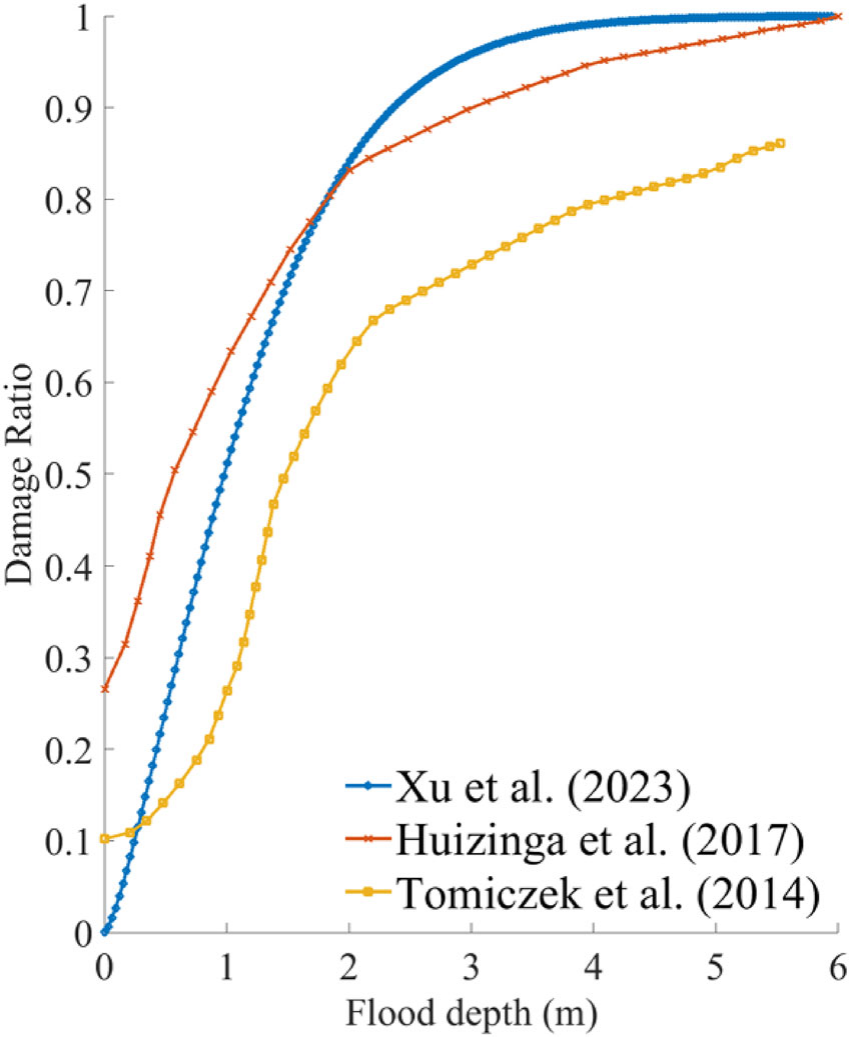
Comparison of residential building damage curves using the data from Xu et al. (2023), Huizinga et al. (2017), and Tomiczek et al. (2014).

To estimate flood damages, each property is matched to its corresponding computational cell on the return period flood map generated by the storm surge model. The flood depth at the property location is assumed to equal the flood depth within that cell. We then compute the damage ratio (DR) for each property using established residential building damage curves, which represent the relationship between flood depth and the DR. Three different damage curves are employed: (1) Xu et al. (2023), a regional damage curve derived from the Hurricane Ike event and the National Flood Insurance Program (NFIP) database, (2) North America global data from Huizinga et al. (2017), and (3) local FEMA survey data from Tomiczek et al. (2014), which are illustrated in Figure 9. The flood damage for each property is then computed by multiplying the assessed property value by the corresponding DR. If the properties are not flooded, DR is set to 0, although the DR is greater than 0 with zero flood depth under the curves of Huizinga et al. (2017) and Tomiczek et al. (2014). For the future scenarios, DR is set to 1 for the properties submerged by SLR, and the flood damage caused by SLR is separated from the damage caused by storm surge to isolate the impact of the Ike Dike on storm surge risk. Finally, the total flood damage is calculated as the sum of the flood damages for all properties.

#### 2.3.2 Expected annual damages

To quantify flood risk, the expected annual damage (EAD) is used as an estimate of the risk integrated over a range of return periods (Arnell, 1989). EAD can be approximated using the mid‐range method as follows:

$$\mathrm{EAD}=\sum_{i=0}^{N}\left(P_{i}-P_{i+1}\right)\frac{D_{i+1}+D_{i}}{2},$$

(12) $$P_{0}=1,\;D_{0}=0,\;P_{N+1}=0,\;D_{N+1}=D_{N}$$

where N is the number of flood events considered, $P_{i}$ is the exceedance probability for the i‐th flood event, and $D_{i}$ is the damage for the i‐th flood event. This method calculates EAD by averaging the damages between successive flood events weighted by the difference in their annual exceedance probabilities. Such the method is sensitive to the selection of probability increments, so we derive EAD using three different sets of return periods for comparison: (1) 10, 25, 50, 100, and 500 years (FEMA, 2013), (2) 5, 10, 25, 50, and 100 years (Arnell, 1989), and (3) 2, 5, 10, 20, 50, 100, 500, 1000, 5000, and 10,000 years (Tariq, 2013).

## 3 RESULTS

### 3.1 Probabilistic flood depth predictions

**FIGURE A1 risa70060-fig-0017:**
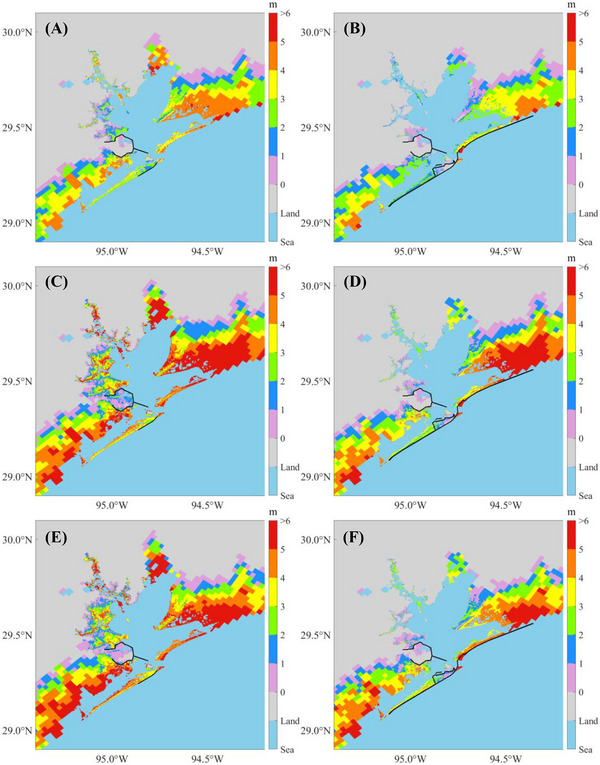
Predictions of 100‐year flood maps under different flooding scenarios for the present climate. The black lines are existing seawalls (for scenarios without the Ike Dike) or the proposed barrier system (for Ike Dike scenarios): (A) Scenario 1; (B) Scenario 2; (C) Scenario 3; (D) Scenario 4; (E) Scenario 5; (F) Scenario 6.

Using the modified GEV distribution, we determined the flood depths for return periods of 2, 5, 10, 20, 50, 100, 500, 1000, 5000, and 10,000 years for each flooding scenario. Figure A1 represents the predicted 100‐year flood maps for the present climate (Scenarios 1–6), whereas Figure A2 shows the predictions for the future climate (Scenarios 7–24). The results indicate that synthetic storm tracks downscaled from the GFDL model generate the largest floods in terms of depth and flooded area, followed by those from the HadGEM, with the smallest floods produced by the CanESM, regardless of whether the scenario pertains to the present or future climate. For the future scenarios, Chambers County, TX, exhibits the largest submerged area due to SLR, followed by Galveston County, TX, which includes Galveston Island and Bolivar Peninsula. Harris County, TX, is the least affected by SLR.

Comparing flood maps across scenarios with and without the Ike Dike reveals that the Ike Dike effectively protects the area along Galveston Bay from storm surges. According to Figure 10, the 100‐year flood depth is reduced by up to 5 m under present climate scenarios and by 1–3 m along Galveston Bay and more than 3 m inside the existing Texas City Dike under future climate scenarios. Additionally, Figure 11 shows that most of the reduced flooded areal extent due to the Ike Dike is concentrated around Galveston Bay. However, the Ike Dike does not significantly reduce flood depth in the regions west of Galveston Island and east of the Bolivar Peninsula. In these regions, the flood depth difference does not exceed 2 m under the present climate or 1 m under the future climate (Figure 10). In some areas, the flood depth is even higher with the Ike Dike than without it, as surges that cannot pass through the Ike Dike accumulate outside it, leading to higher surges there.

FIGURE 10Difference in the 100‐year flood depths between scenarios without and with the Ike Dike for each flooding scenario. The black solid lines are existing seawalls and the proposed barrier system, and the black dashed line is the coastline under present conditions: (A) Scenarios 1 and 2; (B) Scenarios 3 and 4; (C) Scenarios 5 and 6; (D) Scenarios 7 and 8; (E) Scenarios 9 and 10; (F) Scenarios 11 and 12; (G) Scenarios 13 and 14; (H) Scenarios 15 and 16; (I) Scenarios 17 and 18; (J) Scenarios 19 and 20; (K) Scenarios 21 and 22; (L) Scenarios 23 and 24.

**Figure:**
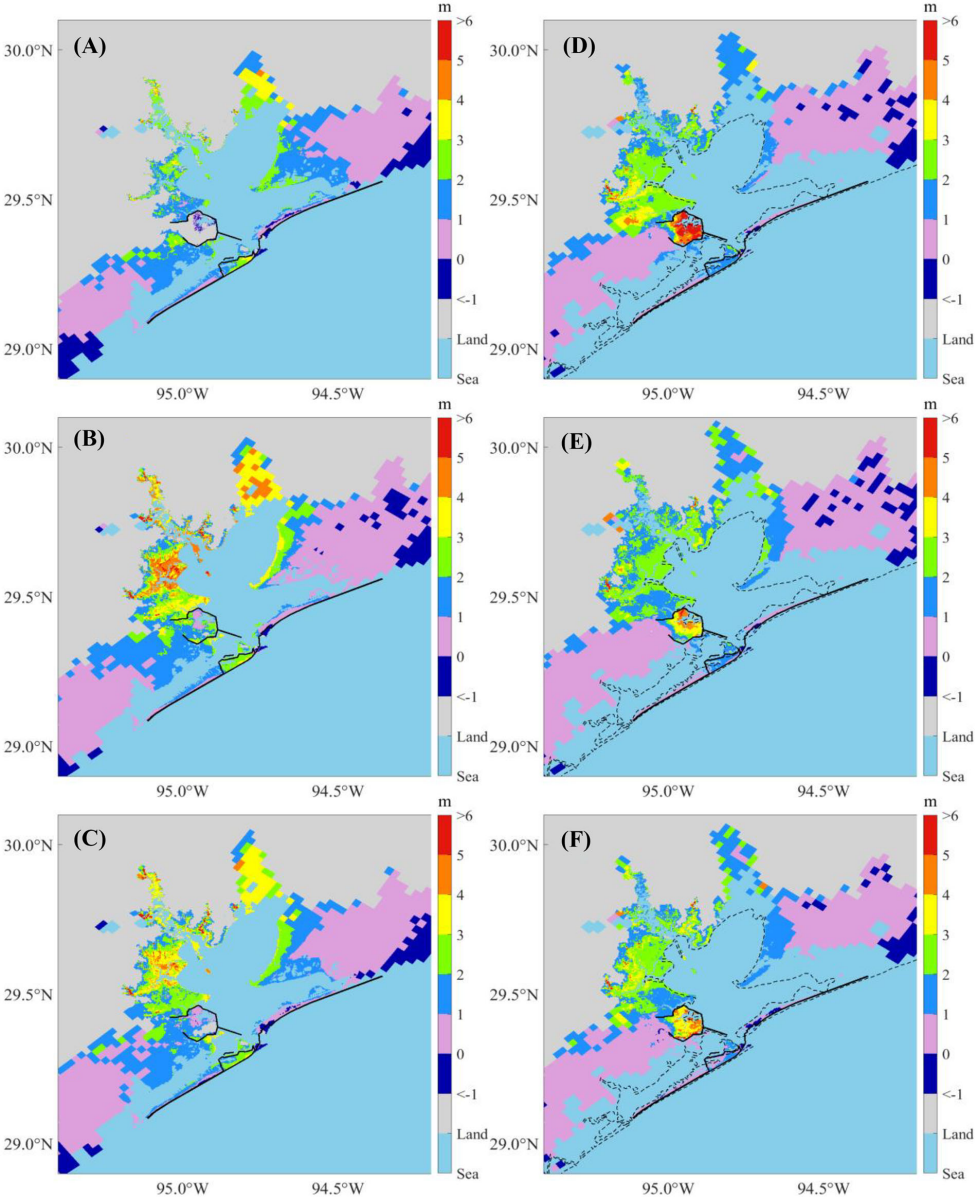

**Figure:**
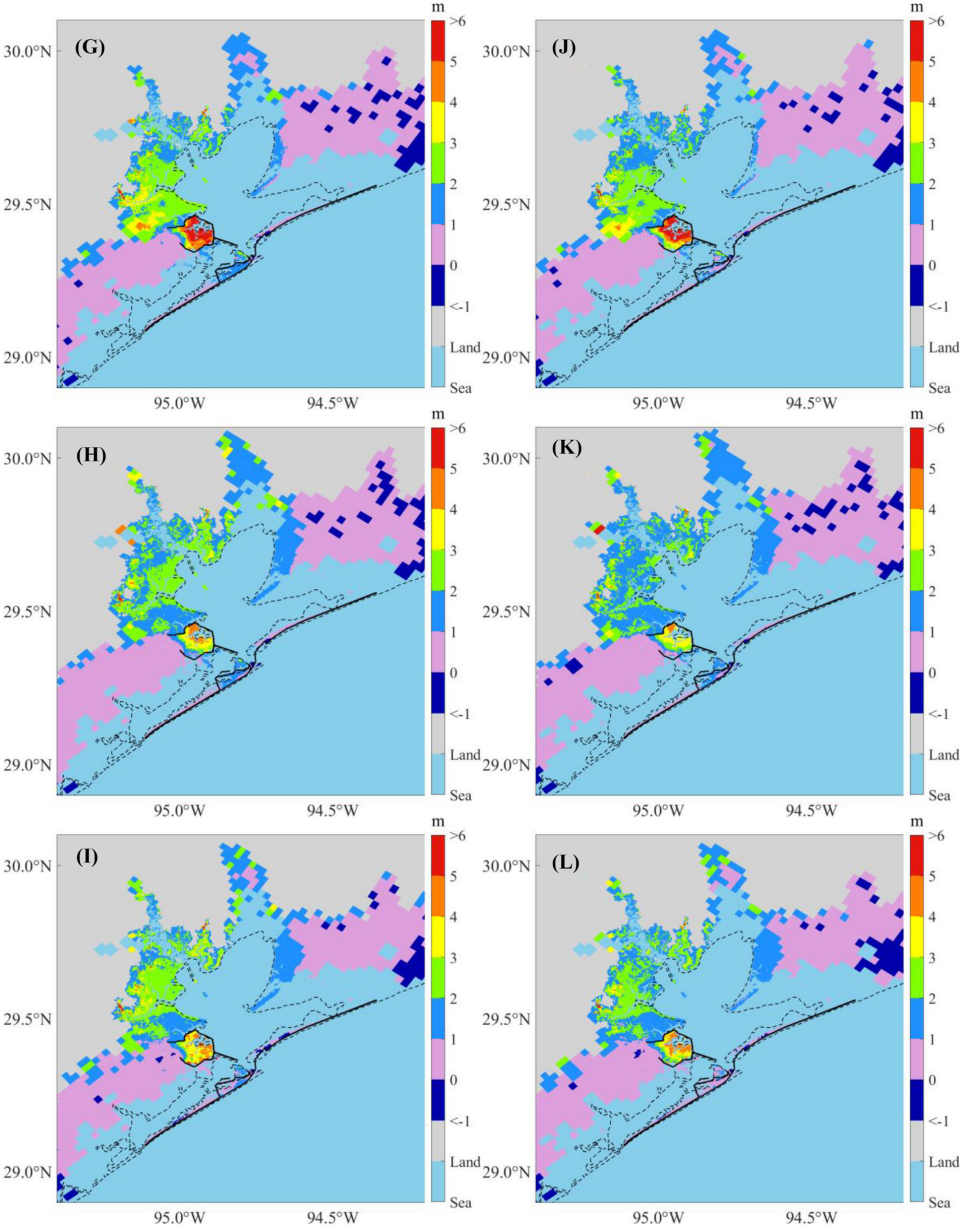

FIGURE 11Flooded areal extent under scenarios without (blue area) and with (pink area) the Ike Dike for each flooding scenario. The black solid and dashed lines are the same as Figure 10: (A) Scenarios 1 and 2; (B) Scenarios 3 and 4; (C) Scenarios 5 and 6; (D) Scenarios 7 and 8; (E) Scenarios 9 and 10; (F) Scenarios 11 and 12; (G) Scenarios 13 and 14; (H) Scenarios 15 and 16; (I) Scenarios 17 and 18; (J) Scenarios 19 and 20; (K) Scenarios 21 and 22; (L) Scenarios 23 and 24.

**Figure:**
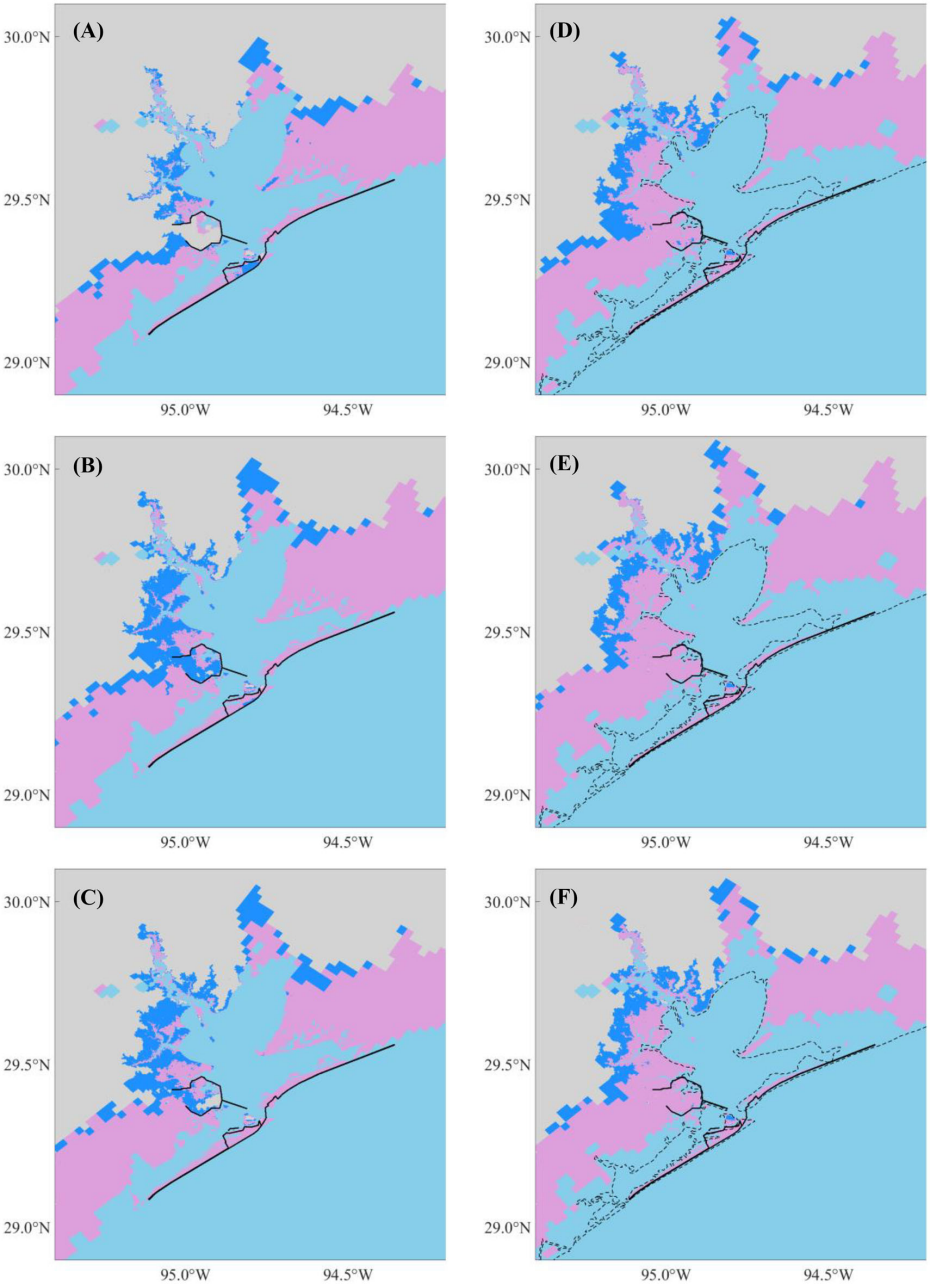

**Figure:**
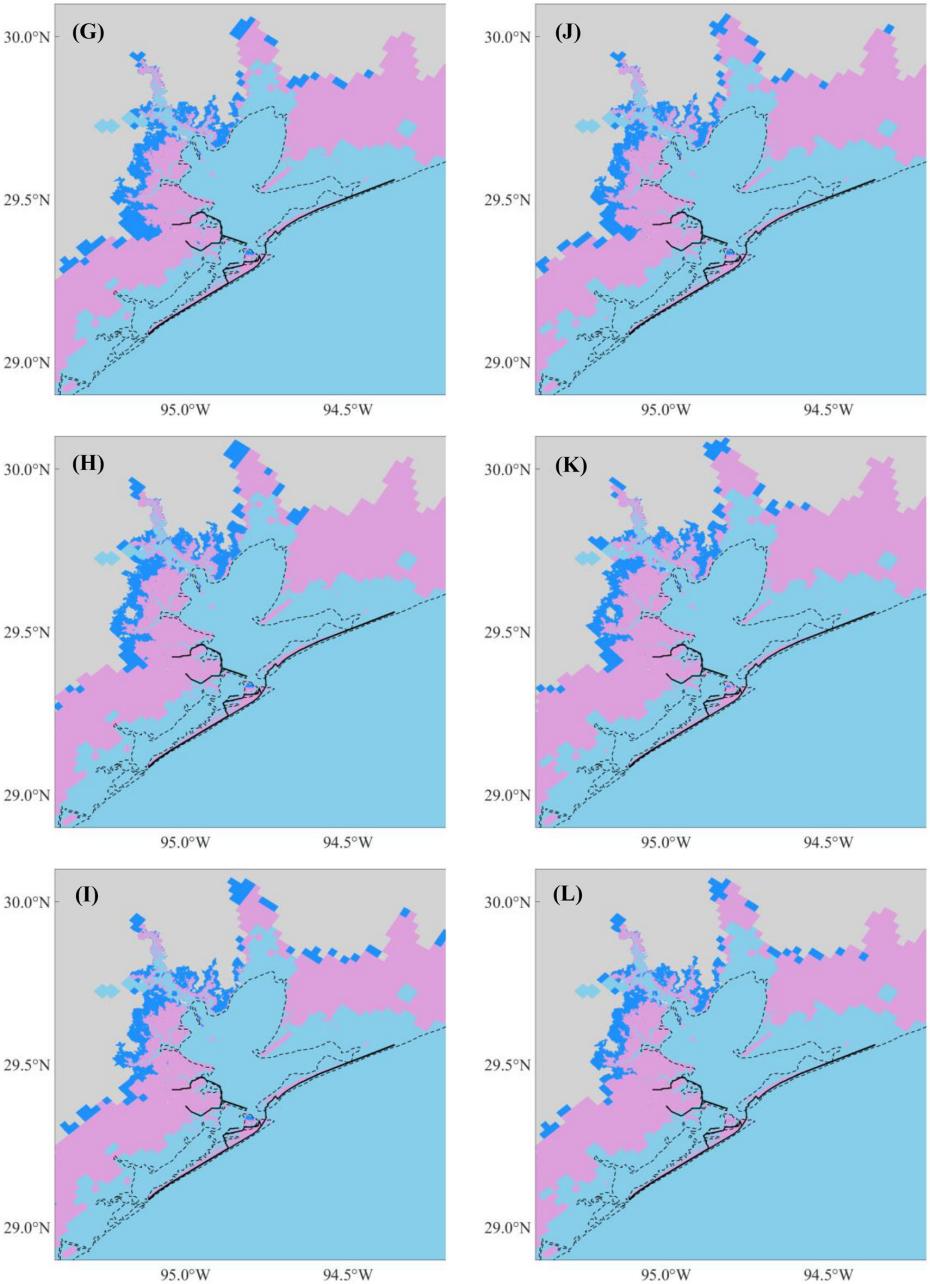

In the entire HGA, the area‐weighted average flood depth is reduced by 1–1.25 m under the present climate, which represents a reduction of 35%–37% compared to the depth without the Ike Dike. The flooded areal extent under the present climate is reduced by 520–800 km^2^, which is 6%–6.5% of the original flooded area. For future climate scenarios, the difference in area‐weighted average flood depth is 0.90–1.05 m, for a reduction of 26%–32.5%. The difference in the flooded areal extent is 455%–585 km^2^, accounting for a reduction of 3.8%–5.1%. These results indicate that the Ike Dike can reduce both flood depth and flooded area more effectively for present climate scenarios compared to future climate scenarios. These results for area‐weighted average flood depth and flooded areal extent are listed in Table 4.

**TABLE 4:** Difference in area‐weighted average 100‐year flood depth and flooded areal extent between scenarios without and with the Ike Dike for each flooding scenario.

Present/Future	SLR scenario	GCM	(Difference) = (No Ike Dike)—(Ike Dike) [Percent reduction]	
			Area‐weighted average flood depth	Flooded areal extent
Present	–	CanESM	1.015 m	521.32 km^2^
			**[37.00%]**	**[6.40%]**
		GFDL‐6.0	1.251 m	803.14 km^2^
			**[35.29%]**	**[5.98%]**
		HadGEM‐6.0	1.245 m	791.24 km^2^
			**[36.40%]**	**[6.25%]**
Future	SLR Scenario 1 (0.99 m)	CanESM	1.047 m	584.08 km^2^
			**[31.35%]**	**[5.01%]**
		GFDL‐6.0	1.001 m	546.51 km^2^
			**[28.85%]**	**[4.21%]**
		HadGEM‐6.0	1.064 m	536.89 km^2^
			**[32.59%]**	**[4.97%]**
	SLR Scenario 2 (1.08 m)	CanESM	1.019 m	544.49 km^2^
			**[30.58%]**	**[4.74%]**
		GFDL‐6.0	0.976 m	531.67 km^2^
			**[28.21%]**	**[4.16%]**
		HadGEM‐6.0	1.051 m	547.73 km^2^
			**[32.44%]**	**[5.12%]**
	SLR Scenario 3 (1.26 m)	CanESM	0.944 m	485.21 km^2^
			**[28.25%]**	**[4.24%]**
		GFDL‐6.0	0.902 m	491.87 km^2^
			**[26.13%]**	**[3.86%]**
		HadGEM‐6.0	0.953 m	455.29 km^2^
			**[29.27%]**	**[4.25%]**

Abbreviations: CanESM, Canadian Earth System Model; GFDL, Geophysical Fluid Dynamics Laboratory; GCM, general circulation models; HadGEM, Hadley Centre Global Environmental Model; SLR, sea level rise.

### 3.2 Flood damages for probability increments

**FIGURE 12:**
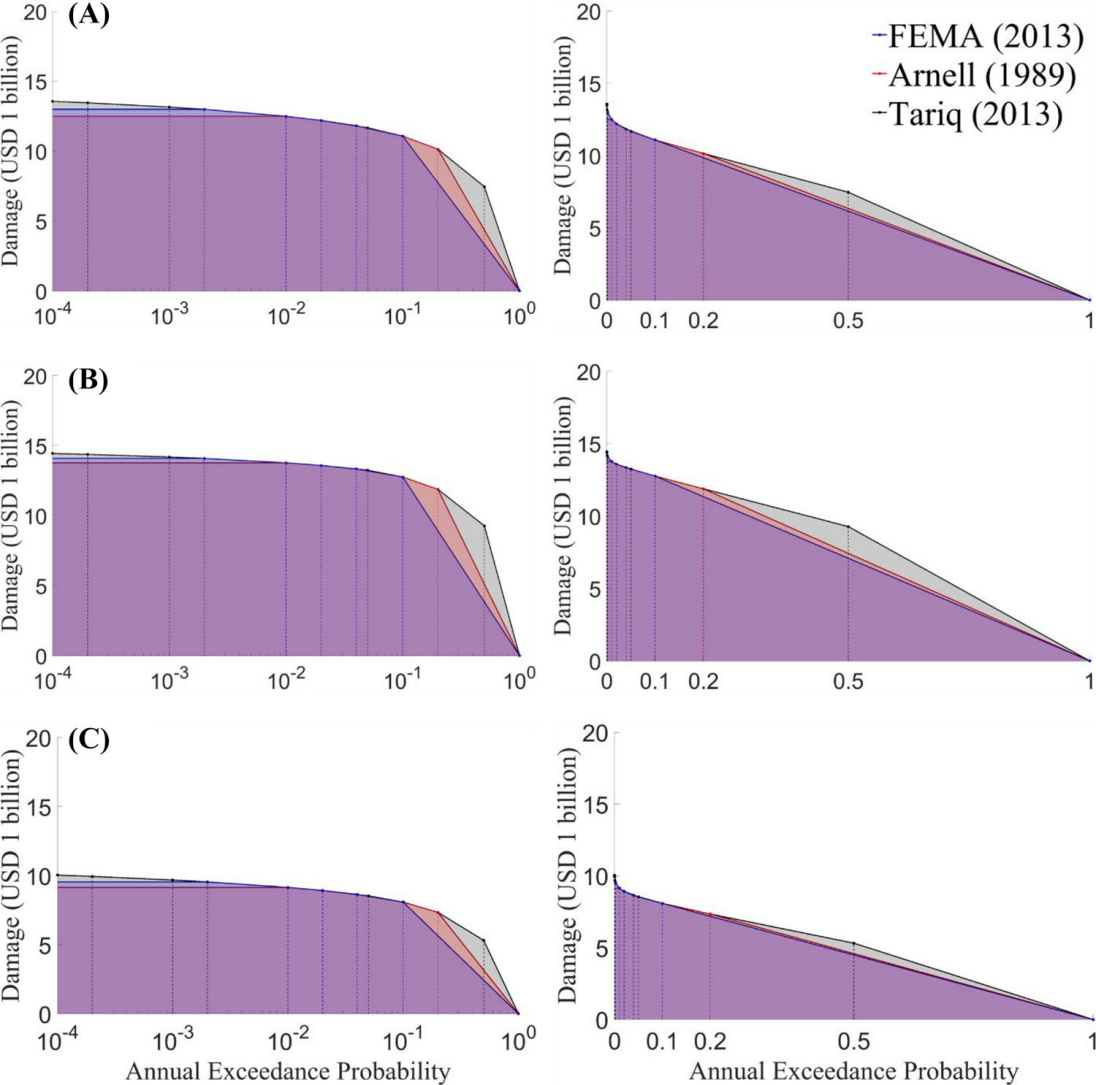
Comparison of total flood damages for specific annual exceedance probabilities under flooding Scenario 1. The left‐side plots use a logarithmic scale *x*‐axis, whereas the right‐side plots use a linear scale *x*‐axis. The shaded area represents the EAD. Damages are estimated using three damage curves: (A) Xu et al. (2023); (B) Huizinga et al. (2017); (C) Tomiczek et al. (2014).

By applying damage curves to the predicted probabilistic flood depths for each residential property, we estimate the total flood damages for each annual exceedance probability. Then the EAD is computed for different sets of probability increments as described in Section 2.3.2. Figure 12 presents the plots of flood damage for specific annual exceedance probabilities using different probability increment sets under flooding Scenario 1. It shows that flood damages from higher annual exceedance probabilities (2, 5, and 10 years) occupy a significant portion of the shaded area, implying their importance in the estimation of EAD. Additionally, a set with a higher number of probability increments provides a more accurate EAD estimate than a set with fewer increments. Therefore, we have chosen to use the set of probability increments from (Tariq, 2013) to estimate EAD for all flooding scenarios.

**FIGURE 13:**
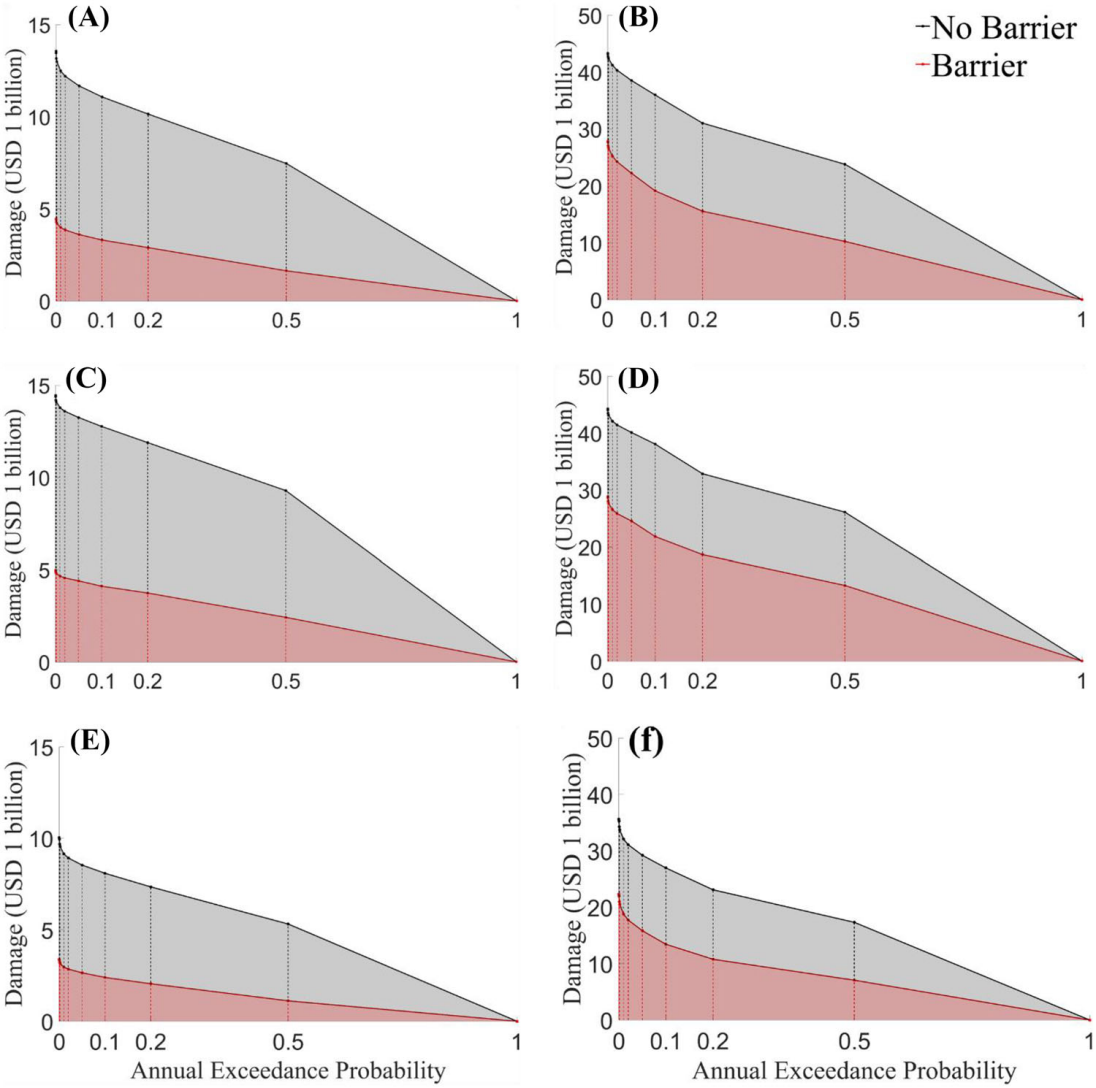
Comparison of total flood damages for specific annual exceedance probabilities under the scenarios without and with the Ike Dike: (A) Scenarios 1 and 2 with Xu et al. (2023); (B) Scenarios 23 and 24 with Xu et al. (2023); (C) Scenarios 1 and 2 with Huizinga et al. (2017); (D) Scenarios 23 and 24 with Huizinga et al. (2017); (E) Scenarios 1 and 2 with Tomiczek et al. (2014); (F) Scenarios 23 and 24 with Tomiczek et al. (2014).

Figure 13 and Table 5 compare the total flood damages under scenarios without and with the Ike Dike (flooding Scenarios 1 and 2, Scenarios 23 and 24) for specific probability increments or return periods. Regardless of damage function applied, the total damage under flooding Scenario 2, with the Ike Dike, is approximately one‐third the damage under Scenario 1, without the Ike Dike. Moreover, flooding Scenario 24, with the Ike Dike, incurs total damage of about 40%–60% compared to Scenario 23, without the Ike Dike. As a result, the Ike Dike is expected to reduce total damage in the HGA by about 40%–70%, which would significantly reduce the risk zone.

**TABLE 5:** Comparison of the total flood damages for specific return periods under flooding Scenarios 1, 2, 23, and 24 (unit: USD 1 billion).

Damage curves	Return period (year)	Flooding scenario #					
		1	2	Percent reduction (%)	23	24	Percent reduction (%)
Xu et al. (2023)	2	7.4642	1.6399	78.03	23.807	10.246	56.96
	5	10.146	2.8996	71.24	31.025	15.551	49.88
	10	11.077	3.3088	70.13	35.983	19.131	46.83
	20	11.677	3.6073	69.11	38.530	22.229	42.31
	50	12.200	3.8542	68.41	40.332	24.235	39.91
	100	12.495	3.9914	68.06	41.197	25.242	38.73
	500	12.993	4.2175	67.54	42.391	26.691	37.04
	1000	13.157	4.2889	67.40	42.702	27.078	36.59
Huizinga et al. (2017)	2	9.2801	2.4197	73.93	26.121	13.295	49.10
	5	11.884	3.7426	68.51	32.869	18.658	43.24
	10	12.763	4.1112	67.79	38.081	21.845	42.64
	20	13.249	4.4008	66.78	40.123	24.571	38.76
	50	13.588	4.5633	66.42	41.440	25.862	37.59
	100	13.776	4.6534	66.22	42.125	26.559	36.95
	500	14.089	4.8010	65.92	43.184	27.687	35.89
	1000	14.191	4.8492	65.83	43.504	28.035	35.56
Tomiczek et al. (2014)	2	5.3163	1.1166	79.00	17.327	7.0646	59.23
	5	7.3442	2.0492	72.10	23.088	10.776	53.33
	10	8.0874	2.3941	70.40	26.961	13.426	50.20
	20	8.5370	2.6495	68.96	29.209	15.822	45.83
	50	8.9248	2.8507	68.06	31.074	17.729	42.94
	100	9.1507	2.9644	67.60	32.076	18.797	41.40
	500	9.5476	3.1534	66.97	33.704	20.493	39.20
	1000	9.6835	3.2149	66.80	34.237	21.012	38.63

### 3.3 Flood risk estimations

The EAD calculated using three damage curves under all flooding scenarios is summarized in Table 6. The EAD varies depending on the synthetic storm data, and the damage curve used, and the SLR scenario. For the present scenarios, the EAD due to storm surges ranges from USD 6.24 to 15.85 billion per year (bn/yr) without the Ike Dike and from USD 2.62 to 6.16 bn/yr with the Ike Dike. For the future scenarios without the Ike Dike, EAD ranges from USD 14.46 to 25.96 bn/yr under SLR Scenario 1, from USD 14.87 to 26.66 bn/yr under SLR Scenario 2, and from USD 15.83 to 28.16 bn/yr under SLR Scenario 3. With the Ike Dike, EAD ranges from USD 5.78 to 14.72 bn/yr under SLR Scenario 1, from USD 6.14 to 15.65 bn/yr under SLR Scenario 2, and from USD 7.27 to 17.25 bn/yr under SLR Scenario 3. The EAD increases as the SLRs and decreases with the presence of the Ike Dike.

**TABLE 6:** Comparison of the expected annual damage (EAD) caused by storm surge and sea level rise (SLR) estimated by different damage curves for each flooding scenario (unit: USD 1 bn/yr).

	Storm surge			
Flooding scenario #	Xu et al. (2023)	Huizinga et al. (2017)	Tomiczek et al. (2014)	SLR
1	8.1281	9.4370	6.2424	0
2	3.1473	3.7434	2.6253	
3	14.060	15.850	10.479	
4	4.5671	5.6864	3.7831	
5	13.197	15.491	9.8559	
6	4.8879	6.1621	3.9471	
7	21.401	22.712	15.935	3.6328
8	8.9297	11.071	6.2996	
9	24.055	25.957	17.946	
10	12.021	14.715	8.4168	
11	19.552	21.211	14.460	
12	8.2012	10.370	5.7848	
13	21.991	23.212	16.419	3.9558
14	9.4539	11.524	6.6800	
15	24.619	26.658	18.469	
16	12.963	15.645	9.0630	
17	20.122	21.753	14.873	
18	8.7786	10.861	6.1373	
19	23.115	24.252	17.384	4.5552
20	10.787	12.905	7.6938	
21	26.038	28.161	19.619	
22	14.595	17.250	10.244	
23	21.400	22.951	15.853	
24	10.406	12.594	7.2691	

Table 7 presents the difference in EAD between scenarios without and with the Ike Dike, illustrating the economic impact of the Ike Dike. The EAD difference ranges from USD 3.62 to 10.16 bn/yr under present scenarios, from USD 8.68 to 12.47 bn/yr under the SLR Scenario 1, from USD 8.74 to 12.54 bn/yr under the SLR Scenario 2, and from USD 8.58 to 12.33 bn/yr under the SLR Scenario 3. For the overall trend in the future, the mean EAD difference across all GCMs and damage curves used is USD 10.824 bn/yr under SLR Scenario 1, USD 10.779 bn/yr under SLR Scenario 2, and USD 10.559 bn/yr under SLR Scenario 3. Although the EAD difference decreases as SLRs, the change in difference is relatively minor.

**TABLE 7:** Difference in expected annual damage (EAD) caused by storm surge between scenarios without and with the Ike Dike for each flooding scenario (unit of difference: USD 1 bn/yr).

			Difference in EAD [Percent reduction]		
Present/Future	SLR scenario	GCM	Xu et al. (2023)	Huizinga et al. (2017)	Tomiczek et al. (2014)
Present	–	CanESM	4.9808	5.6936	3.6171
			**[61.28%]**	**[60.33%]**	**[57.94%]**
		GFDL‐6.0	9.4929	10.163	6.6963
			**[67.52%]**	**[64.12%]**	**[63.90%]**
		HadGEM‐6.0	8.3086	9.3286	5.9088
			**[62.96%]**	**[60.22%]**	**[59.95%]**
Future	SLR Scenario 1 (0.99 m)	CanESM	12.471	11.641	9.6354
			**[58.27%]**	**[51.25%]**	**[60.47%]**
		GFDL‐6.0	12.034	11.242	9.5292
			**[50.03%]**	**[43.31%]**	**[53.10%]**
		HadGEM‐6.0	11.351	10.841	8.6752
			**[58.05%]**	**[51.11%]**	**[59.99%]**
	SLR Scenario 2 (1.08 m)	CanESM	12.537	11.688	9.7390
			**[57.01%]**	**[50.35%]**	**[59.32%]**
		GFDL‐6.0	11.656	11.013	9.4060
			**[47.35%]**	**[41.31%]**	**[50.93%]**
		HadGEM‐6.0	11.343	10.892	8.7357
			**[56.37%]**	**[50.07%]**	**[58.74%]**
	SLR Scenario 3 (1.26 m)	CanESM	12.328	11.347	9.6902
			**[53.33%]**	**[46.79%]**	**[55.74%]**
		GFDL‐6.0	11.443	10.911	9.3750
			**[43.95%]**	**[38.75%]**	**[47.79%]**
		HadGEM‐6.0	10.994	10.357	8.5839
			**[51.38%]**	**[45.13%]**	**[54.15%]**

Abbreviations: CanESM, Canadian Earth System Model; GFDL, Geophysical Fluid Dynamics Laboratory; GCM, general circulation models; HadGEM, Hadley Centre Global Environmental Model; SLR, sea level rise.

Absolute values of EAD difference are higher under future scenarios than under present scenarios. On the other hand, the percent reduction of the EAD is 58%–68% under present scenarios, which is higher than under future scenarios. The future scenarios incur a percent reduction of 43%–60% under SLR Scenario 1, 41%–59% under SLR Scenario 2, and 39%–56% under SLR Scenario 3, showing a clear decreasing trend as SLRs. These results are attributed to the fact that the absolute EAD without the Ike Dike increases with higher SLR values and stronger hurricane intensity. Regardless of the scenario, it is demonstrated that the Ike Dike substantially reduces the EAD in both present and future scenarios, highlighting its effectiveness in mitigating storm surge risks.

## 4 DISCUSSION

### 4.1 Sensitivity of storm surge risk to damage curve

To evaluate the variability of EAD with respect to different damage curves, we compute the coefficient of variation (CV) of EAD for each flooding scenario as follows (Table 8):

(13) $$\delta =\frac{\sigma }{\mu },$$

where μ and σ are the mean and the standard deviation, respectively, of the three EAD values across three damage curves for each flooding scenario. The present scenarios show CVs around 0.20. For future scenarios without the Ike Dike, the CV ranges from 0.17 to 0.20, whereas it ranges from 0.25 to 0.29 with the Ike Dike. These results indicate that the presence of the Ike Dike has a minimal impact on the sensitivity of EAD in the present scenarios, whereas EAD is more sensitive to the damage curve when the Ike Dike is considered in the future. It suggests that the selection of the damage curve in future scenarios becomes more critical when assessing the effectiveness of the Ike Dike on the basis of EAD estimation.

**TABLE 8:** Comparison of the coefficient of variance of expected annual damage (EAD) across different damage curves for each flooding scenario and the difference between the scenarios without and with the Ike Dike.

				Coefficient of variance	
Present/Future	SLR scenario	GCM	Ike Dike	EAD of each scenario	EAD difference
Present	–	CanESM	X	0.2024	0.2215
			O	0.1764	
		GFDL‐6.0	X	0.2031	0.2093
			O	0.2044	
		HadGEM‐6.0	X	0.2206	0.2237
			O	0.2224	
Future	SLR Scenario 1 (0.99 m)	CanESM	X	0.1796	0.1296
			O	0.2726	
		GFDL‐6.0	X	0.1848	0.1171
			O	0.2697	
		HadGEM‐6.0	X	0.1911	0.1381
			O	0.2825	
	SLR Scenario 2 (1.08 m)	CanESM	X	0.1763	0.1267
			O	0.2636	
		GFDL‐6.0	X	0.1834	0.1084
			O	0.2636	
		HadGEM‐6.0	X	0.1901	0.1350
			O	0.2755	
	SLR Scenario 3 (1.26 m)	CanESM	X	0.1706	0.1199
			O	0.2505	
		GFDL‐6.0	X	0.1807	0.1015
			O	0.2521	
		HadGEM‐6.0	X	0.1860	0.1252
			O	0.2653	

Abbreviations: CanESM, Canadian Earth System Model; GFDL, Geophysical Fluid Dynamics Laboratory; GCM, general circulation models; HadGEM, Hadley Centre Global Environmental Model; SLR, sea level rise.

Additionally, we compare the sensitivity to the choice of damage curve and other parameters by computing the CV of EAD across different GCMs (Table 9) and SLR scenarios (Table 10). For the sensitivity to GCM choice, the CV ranges from 0.2 to 0.29 for present scenarios, around 0.1 for future scenarios without the Ike Dike, and around 0.2 with the Ike Dike. These results imply that storm surge risk is more sensitive to the choice of the GCM for present climate scenarios, whereas the damage curve has a greater impact than the GCM for future climate scenarios. Meanwhile, the CVs of EAD across different SLR scenarios are less than 0.05 without the Ike Dike and around 0.1 with the Ike Dike. Therefore, the result is less sensitive to SLR scenario compared to the choices of damage curve and GCM. When comparing the sensitivity of EAD between the scenarios without and with the Ike Dike, the CV of EAD is higher with the Ike Dike than without it for every future scenario, whereas no clear trend appears under present scenarios.

**TABLE 9:** Comparison of the coefficient of variance of expected annual damage (EAD) across different general circulation models (GCMs) for each flooding scenario and the difference between the scenarios without and with the Ike Dike.

			Coefficient of variance		
Present/Future	SLR scenario	Ike Dike	Xu et al. (2023)	Huizinga et al. (2017)	Tomiczek et al. (2014)
Present	–	X	0.2717	0.2651	0.2852
		O	0.2205	0.2465	0.2087
		Difference	0.3080	0.2831	0.2958
Future	SLR Scenario 1 (0.99 m)	X	0.1045	0.1041	0.1086
		O	0.2087	0.1936	0.2041
		Difference	0.0472	0.0356	0.0567
	SLR Scenario 2 (1.08 m)	X	0.1016	0.1055	0.1088
		O	0.2160	0.2045	0.2134
		Difference	0.0523	0.0383	0.0550
	SLR Scenario 3 (1.26 m)	X	0.0997	0.1079	0.1075
		O	0.1942	0.1827	0.1915
		Difference	0.0586	0.0456	0.0618

Abbreviations: SLR, sea level rise.

**TABLE 10:** Comparison of the coefficient of variance of expected annual damage (EAD) across different sea level rise (SLR) scenarios for each future flooding scenario and the difference between the scenarios without and with the Ike Dike.

			Coefficient of variance		
Present/future	GCM	Ike Dike	Xu et al. (2023)	Huizinga et al. (2017)	Tomiczek et al. (2014)
Future	CanESM	X	0.0393	0.0336	0.0445
		O	0.0985	0.0807	0.1046
		Difference	0.0086	0.0160	0.0053
	GFDL‐6.0	X	0.0410	0.0418	0.0458
		O	0.0987	0.0808	0.1003
		Difference	0.0256	0.0153	0.0086
	HadGEM‐6.0	X	0.0456	0.0405	0.0475
		O	0.1252	0.1036	0.1212
		Difference	0.0182	0.0276	0.0088

Abbreviation: CanESM, Canadian Earth System Model; GFDL, Geophysical Fluid Dynamics Laboratory; GCM, general circulation models; HadGEM, Hadley Centre Global Environmental Model.

Overall, the choice of damage curve significantly influences the estimation of EAD depending on flood depth. As shown in Figure 9, the DR provided by different studies varies with flood depth. Xu et al. (2023) show lower DRs compared to Huizinga et al. (2017) and Tomiczek et al. (2014) when flood depths are less than approximately 0.27 m. For depth between 0.27 and 0.18 m, Xu et al. (2023) have lower DRs than Huizinga et al. (2017) but higher than Tomiczek et al. (2014). At depths greater than 1.8 m, Xu et al. (2023) provide the highest DR compared to the other two damage curves. As a result of our EAD estimation described in Section 3.3, we found that the highest EAD was calculated using Huizinga et al. (2017), followed by Xu et al. (2023), and the lowest using Tomiczek et al. (2014). This pattern aligns with the range of flood depths from 0.27 to 1.8 m, suggesting that most of the vulnerable properties in the HGA are most frequently affected by storm surges with an annual average flood depth within this range. Approximately 90% of the residential properties in this study are located in Harris County, TX, which experiences the least flooding from storm surges within the HGA. The remaining properties are in Galveston County, TX, which also experiences less flooding compared to Chambers County, TX. Consequently, floods with high annual exceedance probabilities, which significantly contribute to overall flood damage, tend to exhibit lower depths.

### 4.2 Impact of sea level rise

**FIGURE 14:**
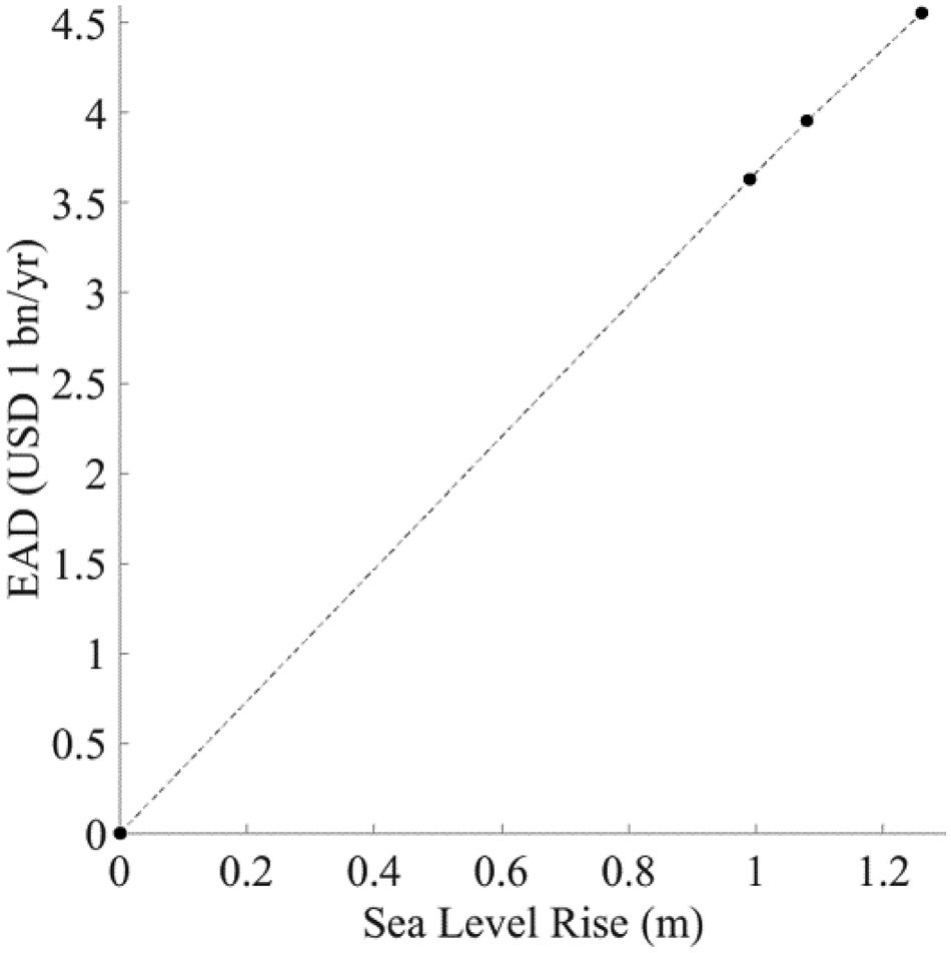
Plot of EAD caused by SLR alone. EAD, expected annual damage.

**FIGURE 15:**
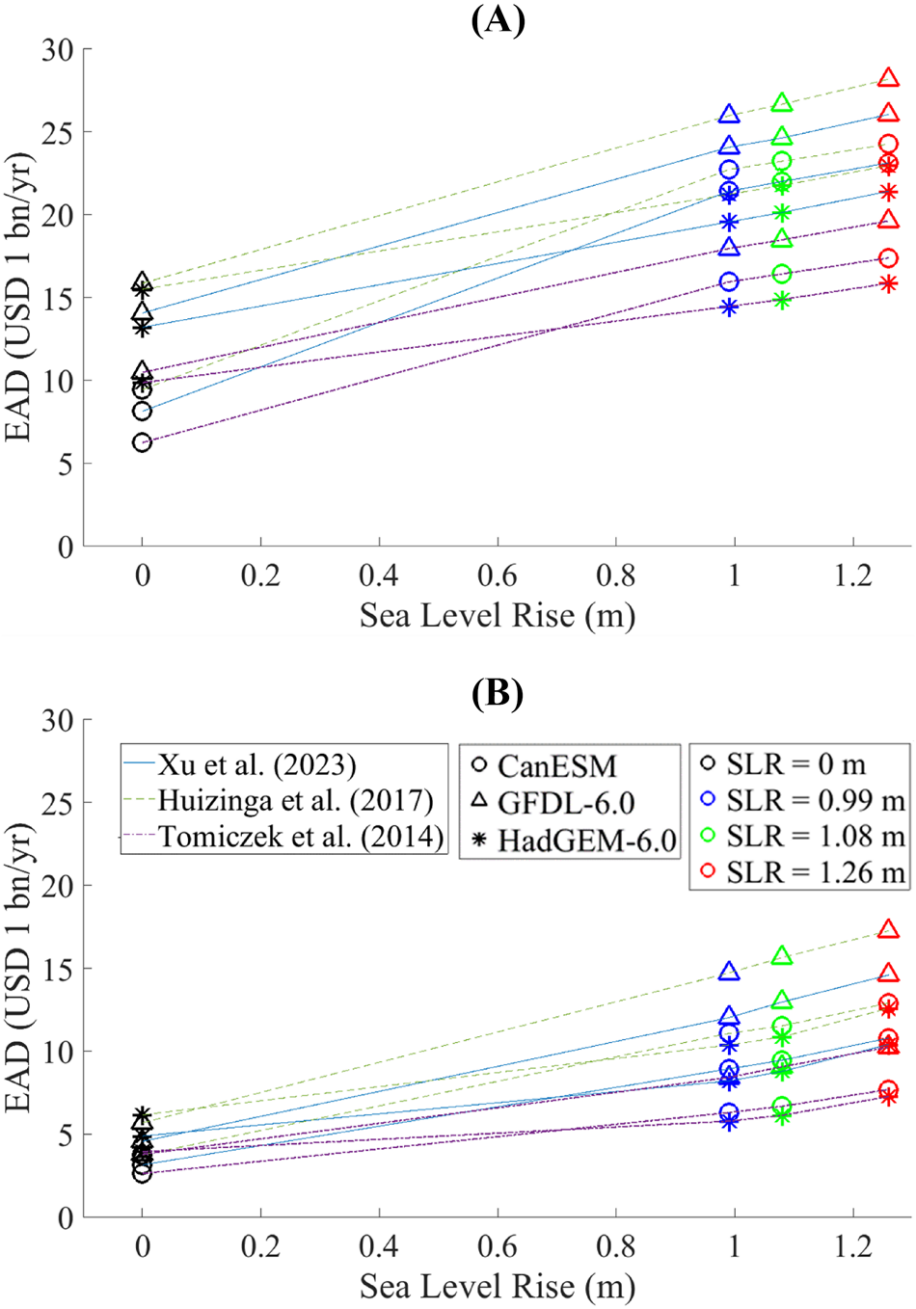
Plot of EAD due to storm surge versus the SLR scenario for different GCMs and damage curves: (A) without the Ike Dike; (B) with the Ike Dike. CanESM, Canadian Earth System Model; EAD, expected annual damage; GFDL, Geophysical Fluid Dynamics Laboratory; HadGEM, Hadley Centre Global Environmental Model.

In the future climate scenarios, the EAD due to SLR alone (no storm surge) is estimated as USD 3.63 bn/yr for SLR Scenario 1, USD 3.96 bn/yr for SLR Scenario 2, and USD 4.56 bn/yr for SLR Scenario 3, as presented in Table 6. These estimates show a linear relationship between EAD and SLR (Figure 14). The EAD values due to SLR are lower compared to those caused by storm surges across all flooding scenarios with the same SLR. This indicates that the direct impact of SLR on flood risk is less hazardous than that of storm surges. However, it is observed that EAD due to storm surges also increases as the SLRs (Figure 15). Although EAD from storm surges is more sensitive to hurricane intensity compared to SLR, as indicated by the variations of EAD attributed to different GCMs and SLR scenarios in Figure 15, this observation highlights the importance of considering SLR in evaluating future storm surge risk.

**FIGURE 16:**
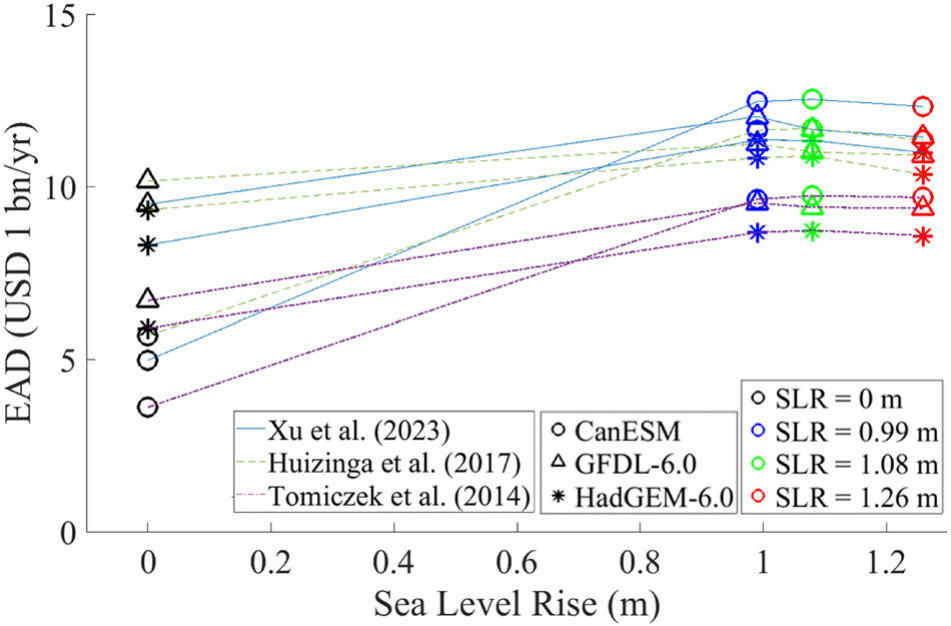
Plot of difference in EAD due to storm surge between the scenarios without and with the Ike Dike. CanESM, Canadian Earth System Model; EAD, expected annual damage; GFDL, Geophysical Fluid Dynamics Laboratory; HadGEM, Hadley Centre Global Environmental Model.

To investigate the impact of SLR on the effectiveness of the Ike Dike, we evaluated the variability of storm surge EAD differences between scenarios without and with the Ike Dike across different SLR scenarios (Figure 16). Our findings conclude that SLR does not have a major impact on the effectiveness of the Ike Dike in reducing storm surge risk, compared to the influence of hurricane intensity and the choice of damage function. Notably, the effectiveness of the Ike Dike is most sensitive to the choice of the damage curve for the future scenarios. This is supported by comparing the CV of EAD differences between scenarios without and with the Ike Dike. The CV across different SLR scenarios ranges from 0.005 to 0.03 (Table 10), which is less than that across different GCMs (0.035–0.06) (Table 9) or damage curves (0.1–0.14) (Table 8).

### 4.3 Cost‐effectiveness of the Ike Dike

We assess the cost‐effectiveness of the Ike Dike using a benefit‐to‐cost (B/C) ratio, considering the Ike Dike feasible if the B/C ratio is greater than 1 (Davlasheridze et al., 2019). The EAD difference is used to represent the benefit, whereas the cost estimate includes construction costs and operation and maintenance costs over 50 years. According to the economic analysis in the USACE's report (USACE & GLO, 2021b), the average annual construction cost is USD 1.077 bn/yr, and the average annual operation and maintenance cost is USD 0.131 bn/yr, amounting to a total average annual project cost of USD 1.208 bn/yr amortized over 19 years, in 2021 dollars. These estimates assume that construction begins in 2025 and ends in 2043, with operations and maintenance continuing for 50 years from 2043 onwards. The analysis used the FY 2021 federal interest rate of 2.5% based on the year of 2043. Table 11 presents the B/C ratio calculations for each flooding scenario. For the present scenarios, the B/C ratio ranges from 2.99 to 8.41, demonstrating the feasibility of the Ike Dike under the present climate and MSL. For the future scenarios, the B/C ratio ranges from 7.23 to 10.38, indicating the Ike Dike is even more economically beneficial under future climate conditions with increased sea levels. These results imply that the Ike Dike is a cost‐effective solution for mitigating storm surge risks under both current and future conditions.

**TABLE 11:** B/C ratios over 50 years for each flooding scenario.

Present/Future	SLR scenario	GCM	Xu et al. (2023)	Huizinga et al. (2017)	Tomiczek et al. (2014)
Present	–	CanESM	4.1232	4.7132	2.9943
		GFDL‐6.0	7.8579	8.4131	5.5433
		HadGEM‐6.0	6.8780	7.7222	4.8914
Future	SLR Scenario 1 (0.99 m)	CanESM	10.3237	9.6366	7.9763
		GFDL‐6.0	9.9619	9.3063	7.8884
		HadGEM‐6.0	9.3965	8.9743	7.1815
	SLR Scenario 2 (1.08 m)	CanESM	10.3783	9.6755	8.0621
		GFDL‐6.0	9.6490	9.1167	7.7864
		HadGEM‐6.0	9.3899	9.0166	7.2315
	SLR Scenario 3 (1.26 m)	CanESM	10.2053	9.3932	8.0217
		GFDL‐6.0	9.4727	9.0323	7.7608
		HadGEM‐6.0	9.1010	8.5737	7.1059

Abbreviations: CanESM, Canadian Earth System Model; GFDL, Geophysical Fluid Dynamics Laboratory; GCM, general circulation models; HadGEM, Hadley Centre Global Environmental Model; SLR, sea level rise.

However, the estimated B/C ratios are much higher compared to the values reported by USACE and GLO (2021b). The equivalent annual net benefits for residential and commercial structures are USD 1.529 bn/yr under the low SLR scenario (0.43 m by 2085), USD 1.959 bn/yr under the intermediate SLR scenario (0.64 m by 2085), and USD 3.320 bn/yr under the high SLR scenario (1.34 m by 2085). This results in B/C ratios of 1.266, 1.622, and 2.748, respectively (Table 12). The USACE's study utilized over 120 events of historical hurricanes and tropical storms from the year 1851 to the present, which have significantly lower intensity and frequency compared to the synthetic hurricanes used in this study. The synthetic tracks were statistically downscaled from GCMs under the SSP5‐8.5 scenario, which is very conservative, meaning it represents the worst possible case or extreme hazard scenario with respect to both storm intensity and SLR. Our findings indicate that coastal flood risk and the effectiveness of the coastal barrier are more sensitive to storm intensity than to SLR. Consequently, the B/C ratio is also more affected by the variance of the climatological variables. Moreover, our scenario is the most conservative among the SSP‐based scenarios projected by the IPCC, suggesting that the B/C ratio estimates would align more closely with those from the USACE's analysis if a less conservative scenario were used. Therefore, our results represent an upper bound for estimates of flood damage and risk.

**TABLE 12:** Total equivalent annual damages to residential and commercial structures and net benefit scenarios reported by the USACE and GLO (2021b) (unit of damages, benefits, and costs: USD 1 bn/yr).

	Equivalent annual damages				
SLR scenario by 2085	No Ike Dike	Ike Dike	Equivalent annual benefits	Total average annual costs	B/C ratio
Low (0.43 m)	$2.310	$0.781	$1.529	$1.208	1.266
Intermediate (0.64 m)	$3.328	$1.369	$1.959		1.622
High (1.34 m)	$7.735	$4.415	$3.320		2.748

Abbreviations: SLR, sea level rise.

Although the B/C ratio provides a quantified metric that justifies the feasibility of the Ike Dike, it should not be the sole criterion for deciding on the construction of the system. The B/C ratio is based on the difference in EAD, which means it does not fully capture the absolute flood damage that could still occur from storm surges in the HGA even with the Ike Dike in place. If storm surge risk is still significant and severe to the area with the Ike Dike, an improved plan may be required to protect the HGA, even if the B/C ratio is high enough. This suggests considering not only the relative economic impacts but also the absolute flood risk with the presence of the Ike Dike. Furthermore, this study's benefit estimation only considered residential properties in Harris and Galveston Counties, TX. It does not include commercial, industrial, or agricultural properties and lands, which are also significant contributors to the region's overall flood risk. Especially, properties and lands in Chambers County, TX, are not included in this cost‐effectiveness analysis, whereas this area is mostly vulnerable to flooding from storm surges and SLR. Additionally, the USACE's plan includes not only the Ike Dike barrier system along Galveston Island and Bolivar Peninsula but also a bay defense system along Galveston Bay, which incorporates lake and bay gate systems as well as nonstructural improvements (USACE & GLO, 2021a). These two proposed systems likely have interdependent effects on protecting the HGA. However, this study focuses solely on the Ike Dike barrier system and does not consider the bay defense system, which may influence the overall B/C analysis. Therefore, the complete economic impact of the Ike Dike on the HGA is not fully represented.

Importantly, cost‐benefit analysis while helping to validate the efficacy and feasibility of the proposed coastal spine, overlook equity considerations related to the differential impact (in terms of risk reduction) this project may have on homeowners with different socioeconomic and demographic backgrounds or on neighborhoods differing by social and economic makeup (Hahn, 2021; Martens, 2011). The analysis conducted here was a simple, traditional one, considering only economic value, thereby neglecting the indirect costs incurred by disaster impacts on health and livelihoods. Moreover, the economic analysis does not address the ramifications of the Ike Dike on related issues of cultural importance and social capital. Nor has this study explored how public support for the Ike Dike (e.g., (Ross & Atoba, 2022))—a factor critical for securing and sustaining funding for the mitigation project—may change with consideration of future SLR and changing climate conditions. Critically, our analysis did not factor in future development patterns and subsequent land‐use change in the HGA, which will undoubtedly have implications for the efficiency and feasibility evaluations of the proposed coastal barrier system. These factors suggest that a comprehensive socioeconomic reanalysis of the Ike Dike's effectiveness is recommended in the future, especially if storm surge risk remains high despite the storm surge barrier system.

## 5 CONCLUSION

In this study, we validated a storm surge model against the data from Hurricane Ike and then used this model to estimate the probabilistic risk of storm surge flooding in the HGA. Flood maps were produced from storm surge simulation for different scenarios, considering both the presence and absence of the Ike Dike. These scenarios incorporated data from several GCMs and different SLR projections. Statistical downscaling of the GCMs generated synthetic tracks of hurricanes that significantly impact the HGA with storm surges. The hydrodynamic model simulated these tracks and produced flood maps for each hurricane. Annual maxima of these flood maps were used to perform extreme value analysis to construct flood maps for specific annual exceedance probabilities. By applying building damage curves on each residential property with the corresponding flood depth, flood damage was estimated for a given return period. Finally, flood risk was quantified by EAD under different flooding scenarios, offering insights into how the Ike Dike might change flood risk under various conditions of climate change and SLR. We found that the Ike Dike would reduce probabilistic flood depth and flooded areal extent behind the barrier under both present and future conditions, leading to the reduction in flood damage on the residential properties in the HGA. This reduction mitigated the storm surge risk, demonstrating the feasibility of the Ike Dike by providing B/C ratios greater than 1 for all flooding scenarios.

However, we observed a wide range of EAD values, which varied depending on the SLR scenarios, GCMs, and residential building damage curves used. The sensitivity analysis of the EAD to these input parameters found that the EAD was most sensitive to the GCM for present climate scenarios and to the choice of damage curve for future climate scenarios, while being least sensitive to SLR scenarios for both present and future climate scenarios. This trend was consistent with the EAD difference that represents the effect of the Ike Dike on mitigating storm surge risk. This suggests that the choice of climate model (related to hurricane intensity and frequency) and the choice of damage function are critical factors in evaluating storm surge risk, as well as the performance of the coastal defense, whereas they remain robust across different SLR scenarios for future climate scenarios.

Under future conditions, although the direct flood risk due to SLR is less than the risk from storm surges, the overall storm surge risk is amplified by the increased sea level. Nonetheless, the effectiveness of the Ike Dike is only marginally impacted by SLR. The selection of the properties to be used was also crucial to the risk analysis. The study used only residential properties located in Harris and Galveston Counties, TX, but Chambers County, TX, presented greater flood depth and flooded area, which is likely to significantly impact the flood risk in the HGA. Further study should pursue a more comprehensive analysis, in terms of property type and socioeconomic factors considered, of the effectiveness of the Ike Dike.

## Data Availability

The data that support the findings of this study are available on request from the corresponding author. The data are not publicly available due to privacy or ethical restrictions.

## APPENDIX A PREDICTIONS OF 100‐YEAR FLOOD MAPS

### A.1

[Fig risa70060-fig-0017], [Fig risa70060-fig-0018]
